# A Linear Topology-Aware Alignment Framework for Belt Conveyor Maintenance Knowledge Graph

**DOI:** 10.3390/s26185833

**Published:** 2026-09-14

**Authors:** Xin Li, Yutong Wang, Cong Han, Ziming Kou

**Affiliations:** 1 College of Robotics Science and Engineering, Taiyuan University of Technology, Taiyuan 030024, China; 2 Shanxi Provincial Engineering Laboratory for Mine Fluid Control, Taiyuan 030024, China

**Keywords:** belt conveyor, spatiotemporal knowledge graph, large language model, linear topology alignment, predictive maintenance, fault tracing

## Abstract

To address spatial confusion and semantic collapse in maintenance knowledge graph construction for long-distance belt conveyor systems, this study proposes a linear topology-aware alignment framework driven by large language models. The framework constructs a Physical–Space–Fault–Rule ontology and introduces a normalized linear topology mapping mechanism to assign unique spatial anchors to highly homogeneous components. For noisy and colloquial maintenance logs, a Condition–Constraint–Action–Space (CCAS) extraction mechanism is developed to reconstruct implicit fault causal chains and spatial information from unstructured text. To prevent erroneous merging of components with similar semantic descriptions but different physical locations, the LTA-Alignment algorithm integrates Gaussian topological penalties with LLM-based grey-zone arbitration. A structured gold-standard annotation protocol is further established using maintenance logs, BOM records, spatial anchors, and closed-loop repair evidence. Experiments over three predefined runs show that CCAS achieves a global F1-score of 94.10 ± 0.56%, exceeding T5 by 6.20 percentage points. LTA-Alignment achieves a Merge F1-score of 95.75 ± 0.53% and an entity cluster purity of 96.70 ± 0.53%, exceeding SCSA by 3.36 and 3.90 percentage points, respectively. In downstream fault traceability, the dynamic spatiotemporal knowledge graph achieves an Accuracy@1 of 92.78 ± 0.36%, 10.16 percentage points higher than R-GCN, together with an estimated 68.50 ± 0.73% reduction in rule-based simulated troubleshooting time relative to Keyword Retrieval. The results demonstrate that combining normalized spatial topology with LLM reasoning improves entity disambiguation, fault-causal reconstruction, and traceability performance under the evaluated non-branching belt conveyor maintenance setting.

## 1. Introduction

In the context of the Industrial Internet of Things and intelligent manufacturing, belt conveyor systems serve as critical infrastructure for bulk material transport in coal, metallurgy, power generation, and port operations. Their operational stability and safety directly determine production efficiency and economic performance across the entire industrial chain [1]. With the advent of Industry 4.0, digital twins, predictive maintenance, and intelligent fault diagnosis technologies are increasingly integrated into traditional asset management systems in mines and factories [2]. However, long-term industrial practice reveals a persistent data-silo problem in conveyor maintenance management. Enterprises possess detailed and well-structured technical documentation, including equipment manuals, bills of materials, and standardized operating procedures [3], whereas information reflecting real-time degradation and emerging fault characteristics is dispersed across inspection logs, shift reports, and maintenance work orders recorded in noisy field environments. Produced by frontline inspectors and technicians, these records are highly unstructured and contain domain-specific abbreviations, local technical jargon, spelling inconsistencies, and complex fault-evolution patterns [4]. Conventional relational database-based asset management systems can record component replacement and spare-part information, but provide limited support for identifying repeated failures at specific locations or representing how local defects propagate through the physical structure [5]. This semantic gap severely limits the transition from reactive maintenance to intelligent predictive maintenance in belt conveyor systems.

Knowledge graphs, as structured semantic networks that represent complex concepts, entities, and their interrelations, have been widely recognized as a core infrastructure for breaking industrial data silos and enabling knowledge automation [6]. By linking fragmented inspection records with formal engineering knowledge, a knowledge graph provides a global reasoning foundation for fault tracing and decision support. The rapid advancement of large language models has fundamentally reshaped the paradigm of industrial information extraction [7]. LLMs leverage large-scale pretraining and strong contextual learning capability, and demonstrate strong ability to extract high-value semantic patterns from noisy and ambiguous text [8]. However, direct application of existing knowledge graph construction frameworks to belt conveyor systems exposes a fundamental theoretical gap. A belt conveyor is not a conventional discrete and centralized system, but a typical long-distance linear asset whose physical structure exhibits extreme one-dimensional spatial extension. A single line often spans several kilometers or even tens of kilometers, and thousands of highly homogeneous physical components are densely distributed along this continuous linear topological manifold. These components share identical specifications, textual descriptions, and local functions [9]. Typical examples include large numbers of carrying idlers and return idlers. A long-distance belt conveyor typically consists of a continuous conveyor belt, drive and tail pulleys, densely distributed carrying and return idlers, and associated drive and protection devices arranged along the conveying route. Maintenance events therefore occur at spatially dispersed physical components and are commonly recorded through component identity, position, abnormal state, and maintenance action. Conventional entity alignment algorithms rely heavily on textual similarity and local graph neighborhood features. Under this paradigm, the first roller in the loading zone and the 800th roller in the unloading zone appear nearly identical in the semantic feature space. Severe homogeneity leads to semantic collapse. The alignment process merges physically isolated components into a single super node [10]. This erroneous fusion destroys spatial resolution. It invalidates subsequent graph-based reasoning on fault propagation [11]. For example, it prevents reliable analysis of whether local jamming-induced frictional heating can propagate along the belt direction and trigger misalignment risk.

Across existing information extraction, industrial knowledge graph, entity alignment, and spatiotemporal reasoning methods, four challenges remain prominent for long-distance linear conveying equipment: spatial information in maintenance text is weakly constrained, continuous linear topology is insufficiently represented, homogeneous components are difficult to distinguish by semantic features alone, and fault-tracing models depend strongly on structured sensor data. These coupled challenges motivate the integration of text understanding, continuous spatial anchoring, topology-aware entity alignment, and dynamic fault reasoning within a unified framework.

Accordingly, a large language model-driven framework is proposed for constructing a topology-aware spatiotemporal knowledge graph for long-distance belt conveyor maintenance. The core idea is to introduce geometric constraints from physical space as a hard complement to semantic space. This strategy enables precise disambiguation of homogeneous components. The main contributions are summarized in three aspects as follows:

(1) A Physical–Space–Fault–Rule ontology architecture is developed for knowledge modeling of long-distance, non-branching belt conveyor systems. By introducing a normalized linear topology mapping mechanism, highly homogeneous components are assigned unique spatial anchors in a continuous coordinate space. This design extends conventional equipment-centered industrial knowledge graphs from static hierarchical representation to topology-aware spatiotemporal modeling.
(2) A Condition–Constraint–Action–Space (CCAS) extraction mechanism is constructed for noisy maintenance logs. The mechanism uses LLM-based step-by-step structured reasoning to extract abnormal conditions, constraint relations, maintenance actions, and spatial anchors from colloquial field records. It transforms fragmented maintenance text into fault-causal chains with explicit spatial localization, enabling dynamic knowledge evolution from unstructured logs.
(3) A Linear Topology-Aware Alignment algorithm, termed LTA-Alignment, is designed to suppress semantic collapse among homogeneous components. The algorithm combines Gaussian spatial penalties with LLM-based grey-zone arbitration, preventing semantically similar but spatially distant entities from being incorrectly merged while retaining ambiguous cases supported by maintenance context. This improves entity disambiguation and downstream fault traceability.

The remainder of this paper is organized as follows. Section 2 reviews research on large language models for industrial information extraction, industrial knowledge graph construction, and entity alignment. Section 3 presents the proposed theoretical and algorithmic framework. It includes the four-dimensional ontology model, the CCAS extraction mechanism, and the mathematical formulation of the LTA-Alignment algorithm. Section 4 conducts experiments and ablation studies using a large-scale real-and-augmented industrial maintenance text dataset. Section 5 summarizes the conclusions and discusses future applications of spatiotemporal knowledge graphs in multimodal industrial Internet of Things data fusion.

## 2. Related Work

To enable structured modeling and spatiotemporal logic reconstruction of belt conveyor maintenance knowledge, recent advances in generative large language models are systematically reviewed. The focus covers industrial information extraction, knowledge graph construction, entity alignment, and spatiotemporal reasoning. Existing methods are further examined to identify theoretical bottlenecks and technical gaps in linear asset intelligent management scenarios.

### 2.1. Evolution of Large Language Models in Industrial Information Extraction

Information extraction serves as the fundamental basis for domain knowledge graph construction. Its objective is to identify named entities and extract latent semantic relations from large-scale unstructured text. In recent years, large-scale pretrained language models based on generative architectures have fundamentally transformed this paradigm. Meshkin et al. reported that models with massive parameters acquire strong semantic completion and contextual understanding through large-scale pretraining on general corpora. These models support efficient few-shot and zero-shot knowledge extraction under limited labeled data conditions [12].

In engineering applications, Qi et al. proposed a dynamic prompt-based extraction framework built on generative models. The framework demonstrates strong capability in parsing relationships within unstructured and highly colloquial text [13]. To address the challenge of fine-tuning complex industrial instructions, Jin et al. developed a large-scale parameter instruction-tuned model. The model significantly improved the boundary recognition accuracy and fault tolerance of deep networks for industrial long-tail entities [14]. Dagdelen et al., in a study published in Nature Communications, further showed that large language models can leverage chain-of-thought reasoning to extract implicit structured attributes from highly noisy scientific and engineering text. This finding provides strong theoretical support for extracting deep semantic information from unstructured equipment maintenance logs [15].

A review of the above studies shows that large language models achieve superior semantic denoising when processing industrial text with incomplete grammar and mixed technical vocabulary. This capability surpasses that of traditional discriminative models. However, this advantage at the semantic level does not fully meet the requirements of complex physical systems. Most existing extraction methods focus on discrete logical relations or isolated attributes. Few studies incorporate absolute topological constraints of physical components in continuous linear physical space into the extraction prompt design. Li et al. identified this limitation and emphasized that language models without physical coordinate anchoring are prone to logical drift when processing engineering text with strong spatial coupling [16]. This spatial blindness leads to severe positional hallucination during downstream physical validation of extracted knowledge triples. Overall, existing LLM-based extraction methods provide strong semantic parsing capability for noisy industrial text, while their integration with absolute spatial coordinates and equipment-topology constraints remains limited. For long-distance belt conveyors, this limitation reduces the reliability of component localization and spatially consistent fault-causal extraction.

### 2.2. Industrial Knowledge Graphs and Bottlenecks in Linear Asset Modeling

Industrial knowledge graphs aim to eliminate data silos across the full lifecycle of manufacturing systems. They provide a unified semantic representation of hardware assets, maintenance rules, and real-time operational states. Meyers et al. proposed a knowledge graph-based cognitive manufacturing data representation method. Their study demonstrated the structural role of a well-defined ontology in integrating heterogeneous data from industrial Internet of Things environments [17]. In the field of predictive maintenance for heavy equipment, Yan et al. developed an expert ontology-driven asset knowledge graph. This approach organizes strict hierarchical relationships and component dependencies. It enables structured lifecycle management of equipment records [18]. Han et al. investigated knowledge graph reasoning for intelligent fault diagnosis in production lines. Their work highlighted the importance of cross-level causal relationships for rapid root cause identification in complex systems [19]. For automation in knowledge graph construction, Zhong et al. reviewed the evolution of knowledge engineering. The study emphasized that dynamic generation of knowledge triples from multi-source engineering standards will become a key direction for improving industrial efficiency [20].

Despite these advances, the underlying logic of current industrial ontology design remains limited. Mainstream asset knowledge graphs generally adopt highly rigid discrete tree-based containment structures for entity organization. Such architectures perform effectively when modeling centralized discrete equipment, such as CNC machine tools or industrial robotic arms. Li et al. developed an integrated data–model–knowledge representation framework for digital twin railways and emphasized that comprehensive railway management requires the unified modeling of multi-granularity spatial features, spatiotemporal relationships, and interaction processes throughout the asset life cycle [21]. Guo et al. further showed that decentralized and independently managed railway data, analytical models, and domain knowledge have difficulty capturing their dynamic coupling relationships and supporting global association retrieval across multiple spatial scales [22]. These studies reveal the limitations of discrete hierarchical and locally isolated representations in modeling the continuous spatial topology and evolving relationships of long-distance linear assets. The core characteristic of linear assets lies in their one-dimensional topological continuity along extended physical space. Conventional hierarchical tree structures cannot provide a mathematical representation mechanism for continuous linear manifolds. Once the ontology architecture lacks continuous spatial scalar mapping capability, the entire knowledge graph degenerates into a rigid static dictionary. Under such conditions, it becomes incapable of accurately modeling the dynamic cascade propagation of fault hazards, such as abnormal temperature rise or mechanical tearing, along physical topological transmission paths. Overall, existing industrial knowledge graphs are effective for hierarchical equipment organization and heterogeneous information integration, but their representation of continuous spatial topology remains limited. For long-distance belt conveyors, this limitation restricts location-specific component modeling and the representation of fault propagation along the conveyor line.

### 2.3. Entity Alignment Algorithms and Semantic Collapse in Topological Structures

Entity alignment is a critical bottleneck in multi-source knowledge graph fusion and knowledge base updating. Its primary objective is to eliminate redundancy and achieve semantic disambiguation. Traditional deep learning approaches mainly perform entity merging by minimizing feature distances in low-dimensional embedding spaces. Li et al. proposed SCSA, a semi-supervised entity-alignment framework that integrates structural, relational, attribute, and string information to improve entity representation and pseudo-label selection [23]. To overcome the structural heterogeneity of multi-source data, Shi proposed a relation-reflection entity alignment network. The framework enhances cross-graph matching robustness by aggregating multi-order topological information with attention mechanisms [24]. Long et al. further developed an end-to-end adaptive alignment framework, which simplifies the complex integration process of feature extraction across heterogeneous knowledge graphs [25]. In the field of spatial entity alignment, Yang et al. introduced a representation learning method that integrates geographic coordinates with semantic information. This approach significantly improved the alignment accuracy of heterogeneous entities in geographic knowledge graphs [26].

However, when these general alignment algorithms are applied to special industrial assets such as belt conveyor systems, the underlying mathematical assumptions face the risk of failure. Belt conveyors are highly homogeneous linear assets. Thousands of supporting components with identical materials and nearly identical textual descriptions are distributed along the conveyor line. Qiu et al. pointed out that, without rigid constraints imposed by absolute spatial coordinates, heterogeneous graph attention mechanisms alone cannot effectively distinguish equipment nodes that are physically far apart but locally topologically equivalent [27]. Because these components produce almost identical semantic vectors in the embedding space, the alignment process inevitably suffers from catastrophic semantic collapse. As a result, hundreds or even thousands of entities at different physical locations may be incorrectly merged into a single distorted super-node. Overall, existing entity-alignment methods effectively exploit semantic, structural, and relational information, but homogeneous components distributed along a long linear asset remain difficult to distinguish when absolute physical position is weakly constrained. This limitation motivates the incorporation of continuous spatial distance into entity-alignment decisions.

### 2.4. Spatiotemporal Reasoning and Predictive Maintenance in Industrial Scenarios

With the rapid development of spatiotemporal big data mining technologies, integrating temporal evolution and spatial geometric constraints into underlying knowledge graphs has emerged as a new frontier in predictive maintenance. Liu et al. integrated a knowledge graph with a large language model for fault diagnosis in aviation assembly. The framework uses structured domain knowledge to support fault localization and generate interpretable diagnostic information [28]. To address the insufficient utilization of temporal information in existing methods, Zhang developed a time similarity-aware alignment model for dynamically evolving temporal networks. By calculating temporal overlap between entities, the proposed approach significantly improved entity alignment accuracy across cross-period temporal knowledge graphs [29]. In the field of heavy industrial electromechanical equipment, Cai et al. proposed a knowledge-embedded spatiotemporal graph convolutional network. System structural knowledge and sensor spatial location information were encoded into relational triplets and mapped into a low-dimensional embedding space. Experiments conducted on aero-engine and cutting-tool datasets demonstrated the decisive role of spatiotemporal degradation pattern extraction in remaining useful life prediction [30]. For reasoning over multi-relational knowledge graphs, Schlichtkrull et al. proposed the relational graph convolutional network (R-GCN), which performs relation-specific message propagation and supports entity classification and link prediction [31]. By combining an R-GCN encoder with a knowledge graph scoring function, candidate entities can be ranked according to their relational compatibility, providing a general graph-reasoning framework for fault tracing and root-cause retrieval. For high-risk and highly complex environments such as underground coal mines, Sun et al. developed a fine-tuned large language model-based intelligent system. By integrating deep retrieval from domain-specific knowledge bases, the system achieved preliminary temporal perception and autonomous early warning of risk evolution processes [32].

A clear trend emerges from existing studies. Most advanced spatiotemporal models rely heavily on structured, high-frequency sensor data. This assumption limits applicability in harsh bulk material transport environments. Full-line deployment of high-precision sensors is costly. Many critical early fault signals exist only in unstructured maintenance logs. Current research lacks an automated framework that extracts spatiotemporal logic directly from text and maps it to continuous physical coordinates. Overall, existing spatiotemporal reasoning methods provide effective representations of temporal evolution and spatial dependence, but most rely on structured, high-frequency sensor data. In long-distance belt conveyor systems, full-line sensor deployment is costly, while substantial fault-evolution evidence remains embedded in unstructured maintenance logs. Automated extraction of spatiotemporal causal relations from such text and their mapping to continuous physical coordinates therefore remain insufficiently explored.

## 3. Theoretical and Algorithmic Framework

### 3.1. Overall Architecture and Definition of Multi-Source Heterogeneous Data

Daily maintenance management of belt conveyor systems involves multi-source heterogeneous data. In the proposed framework, the physical components and their longitudinal positions define the conveyor topology, engineering rules provide operational constraints, and maintenance logs describe time-varying fault states and handling actions; these elements are mapped respectively into the Physical, Space, Rule, and Fault knowledge dimensions. This characteristic imposes strict requirements on structured modeling. In mathematical form, the industrial data space is defined as a set:

(1) $$\Omega =\left\{D_{design},D_{rule},D_{log}\right\}$$

where *D_design_* denotes the bill of materials and engineering topology. It provides a static structural reference in three-dimensional space. *D_rule_* includes safety operation procedures and industry standards. It defines rigid constraint boundary conditions of the system. *D_log_* denotes unstructured free-text logs recorded by field personnel. It contains temporal clues of equipment degradation and abnormal states.

The core objective of this framework is to construct a highly automated nonlinear mapping mechanism f:Ω→G. This mechanism transforms heterogeneous data into a directed attributed multigraph with spatial logic and causal reasoning capability. The spatiotemporal knowledge graph is formalized as a quadruple:

(2) G=(ε,R,A,Φ)

where ε denotes the entity set, A denotes the multidimensional attribute feature space of entities, Φ denotes the spatial coordinate mapping operator, and R denotes the relation set between entities. The following constraint is satisfied:

(3) $$R\subseteq \left\{\left(e_{h},r,e_{t}\right){|e}_{h},e_{t}\in \varepsilon ,r\in \Gamma \right\}$$

where Γ denotes the predefined set of relation types. Typical relations include hierarchical affiliation, spatial mapping, and fault evolution.

**Figure 1:**
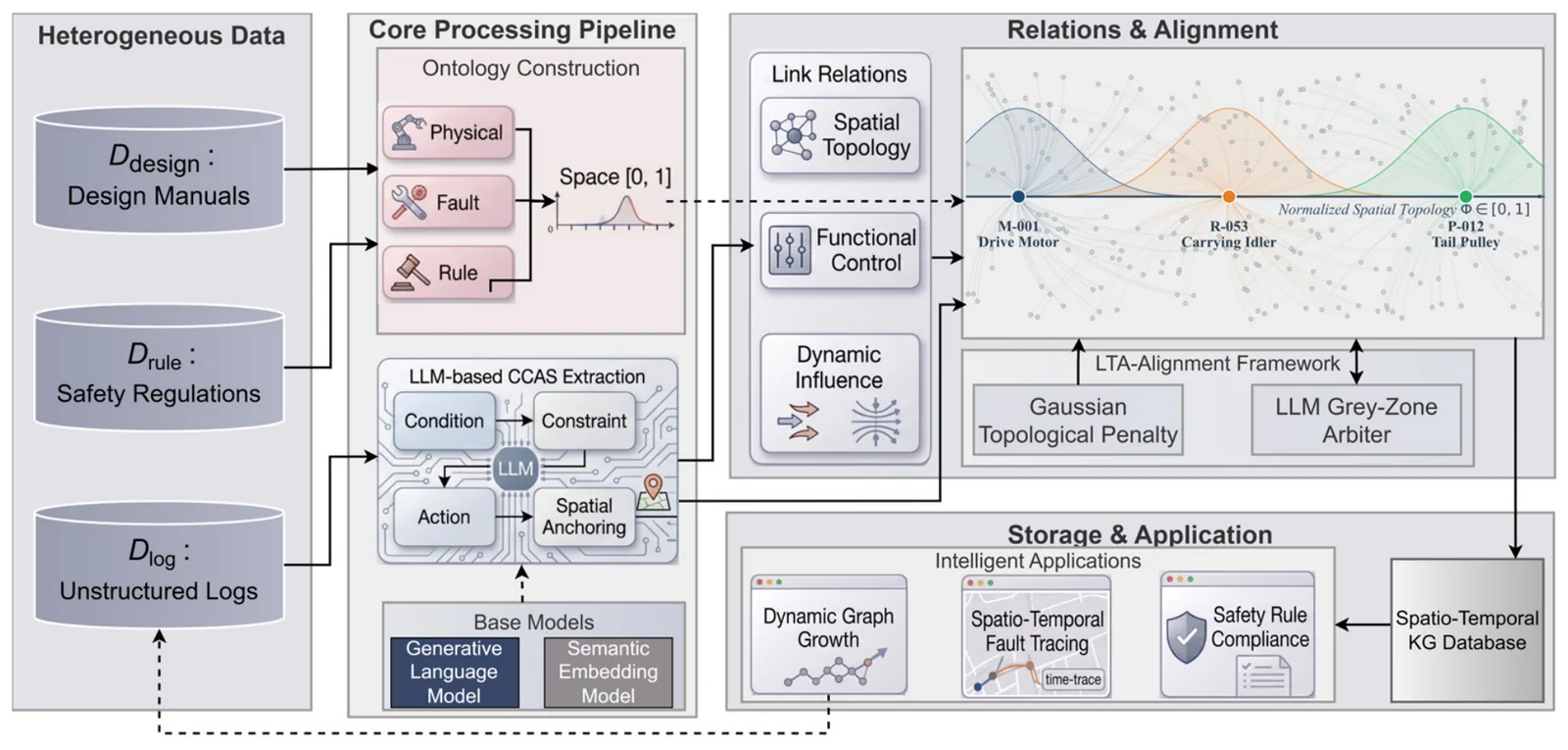
Overall architecture of spatio-temporal knowledge graph construction and evolution.

As illustrated in Figure 1, the proposed system architecture consists of three modules: data ingestion, core processing, and knowledge storage.

During the core processing stage, heterogeneous data from design documents, operational regulations, and unstructured maintenance logs are processed through ontology construction and the LLM-based CCAS extraction mechanism. These data are then uniformly mapped into a predefined spatiotemporal specification framework. Within this process, the Relations & Alignment module visually demonstrates the system’s spatial disambiguation mechanism through a belt-shaped scatter coordinate map. The horizontal axis represents the normalized continuous spatial manifold, Φ ∈ [0, 1]. The overlapping Gaussian distribution curves above the nodes characterize the effective boundary of the topological penalty mechanism in physical space. Through the combined action of this Gaussian constraint and the LLM-based grey-zone arbitration mechanism, the system performs joint verification of topological distance and semantic similarity for scattered candidate entities. Spatially inconsistent matches are eliminated. The resulting structured subgraphs are then imported into the underlying knowledge graph database to support applications such as dynamic graph evolution and spatiotemporal fault tracing.

### 3.2. Four-Dimensional Ontology Design Under Linear Manifold Mapping

Long-distance belt conveyor systems contain large numbers of repetitive components with identical specifications and highly similar maintenance descriptions. This property easily leads to entity ambiguity during knowledge graph construction. To address this issue, a four-dimensional spatiotemporal ontology schema is established. Under the framework of set theory, the global entity space is decomposed into four mutually disjoint subsets:

(4) $$\varepsilon =\varepsilon_{phy}\cup \varepsilon_{flt}\cup \varepsilon_{rul}\cup \varepsilon_{spc}$$

To ensure logical consistency, these subsets satisfy the mutual exclusivity condition:

(5) $$\varepsilon_{i}\cap \varepsilon_{j}=\emptyset ,\forall i\neq j$$

The physical dimension *ε_phy_* includes electromechanical equipment and structural components. The fault dimension *ε_flt_* models abnormal states. The rule dimension *ε_rul_* defines maintenance thresholds. The spatial dimension is modeled as a one-dimensional Riemannian manifold along the conveyor centerline. For the single non-branching conveyor considered in this study, the manifold is endowed with the Euclidean metric, representing the simplest one-dimensional Riemannian case:

(6) $$M^{1}=\left([0,L_{total}],g\right),g=g_{11}(\gamma )d\gamma^{2},g_{11}(\gamma )\equiv 1$$

For a physical component $e_{i}\in E_{phy}$ located at a cumulative centerline distance *l_i_* from the origin, its Riemannian path length is:

(7) $$d_{g}\left(0,l_{i}\right)=\int_{0}^{l_{i}}\sqrt{g_{11}(\gamma )}d\gamma =l_{i}$$

The normalized spatial anchor of *e_i_* is then defined as:

(8) $$\Phi \left(e_{i}\right)=\frac{d_{g}\left(0,l_{i}\right)}{d_{g}\left(0,L_{total}\right)}=\frac{l_{i}}{L_{total}}\in [0,\;1]$$

where $M^{1}$ denotes the one-dimensional spatial manifold defined along the conveyor centerline; *L_total_* is the total centerline length of the conveyor; *g* is the Riemannian metric; $g_{11}(\gamma )$ is its one-dimensional metric component at coordinate γ; and γ is the path coordinate along the conveyor centerline. The function *d_g_*() denotes the Riemannian distance, while $\Phi \left(e_{i}\right)$ denotes the normalized spatial anchor of e*_i_*. Under the Euclidean metric, $g_{11}(\gamma )\equiv 1$, the Riemannian distance reduces to the cumulative centerline distance, and the normalized anchor is therefore calculated as *l_i_*/*L_total_*.

Through this normalization process, physical components with identical semantic descriptions are assigned unique spatial scalar coordinates. It is important to note that, although discrete component identifiers are commonly used in industrial practice, such as 30# belt frame, these discrete symbols exhibit clear limitations in heterogeneous data fusion and graph computation. First, discrete identifiers cannot be directly aligned with absolute physical distances in engineering drawings. Second, irregular spacing between components makes distance estimation based on identifiers lack true metric meaning. This issue leads to the failure of downstream Gaussian kernel-based topological penalty algorithms. Third, local labels cannot generalize topological patterns across conveyor systems of different lengths. In contrast, the normalized continuous manifold mapping proposed in this study constructs a globally consistent absolute scalar coordinate system. This mechanism supports semantic parsing by large language models and remains compatible with discrete identifiers and ambiguous relative position descriptions. It also provides a solid mathematical foundation for subsequent cross-modal spatiotemporal alignment using LTA-Alignment and continuous distance computation.

**Figure 2:**
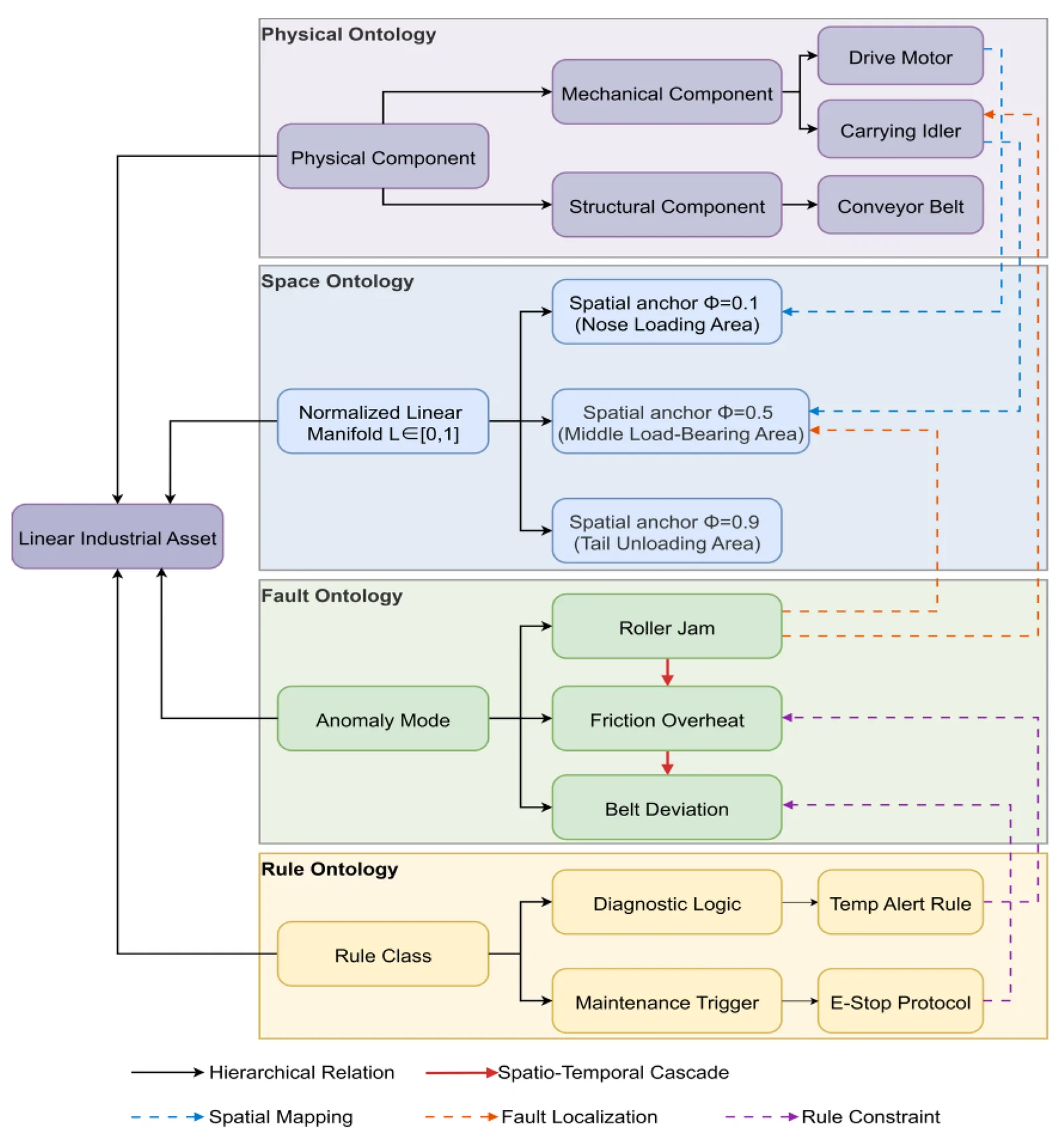
Semantic blueprint of the 4D ontology.

Figure 2 presents a detailed ontological blueprint of the four-dimensional spatiotemporal knowledge graph and its cross-dimensional constraint relationships. 

The ontology consists of four mutually disjoint subsets: Physical, Space, Fault, and Rule. Solid arrows on the left side of the figure define the fundamental hierarchical relationships within each dimension. Cross-dimensional attribute interactions are represented by four types of colored directed dashed lines. The spatial ontology, located at the center, is visualized as a continuous normalized linear manifold. Specific entity components in the physical dimension are strictly projected onto scalar coordinates through Spatial Mapping relations. The blueprint also reveals the spatiotemporal evolution mechanism of abnormal states. Fault nodes are anchored to specific spatial coordinates and form a directed cascading chain such as roller jamming leading to friction overheating leading to belt deviation. In addition, alarm logic and shutdown protocols in the rule dimension impose constraints on specific fault nodes through RuleConstraint relations. This mechanism defines the automatic response boundaries of the system.

### 3.3. Spatiotemporal Fault Extraction Mechanism Based on Language Model Reasoning

Unstructured inspection logs contain numerous non-standard expressions, while equipment failures frequently evolve through cascading processes with temporal and spatial propagation. To represent these characteristics jointly, this study proposes a Condition–Constraint–Action–Space (CCAS) extraction mechanism based on step-by-step structured reasoning by large language models, which maps a natural-language sequence X into a tuple containing four types of features:

(9) $$f_{ccas}:X\to \left\langle C_{cond},C_{constr},A_{act},S_{space}\right\rangle$$

Under the autoregressive generation framework of pretrained language models, a strongly constrained prompt template $P_{ccas}$ is defined. The joint conditional probability distribution of the extracted structured subgraph $G_{sub}$ is expressed as:

(10) $$P\left(G_{sub}|X,P_{ccas}\right)=\prod_{t=1}^{T}P\left(y_{t}|y_{<t},X,P_{ccas};\Theta \right)$$

where *y_t_* denotes the token generated at step *t*, and Θ denotes the parameters of the pre-trained language model. The LLM parameters remain frozen during inference. To characterize the autoregressive generation process under strongly constrained prompt templates, the structured subgraph generation objective is formulated using the negative log-likelihood form:

(11) $$L_{gen}=-\sum_{t=1}^{T}\log P\left(y_{t}|y_{<t},X,P_{ccas};\Theta \right)$$

**Figure 3:**
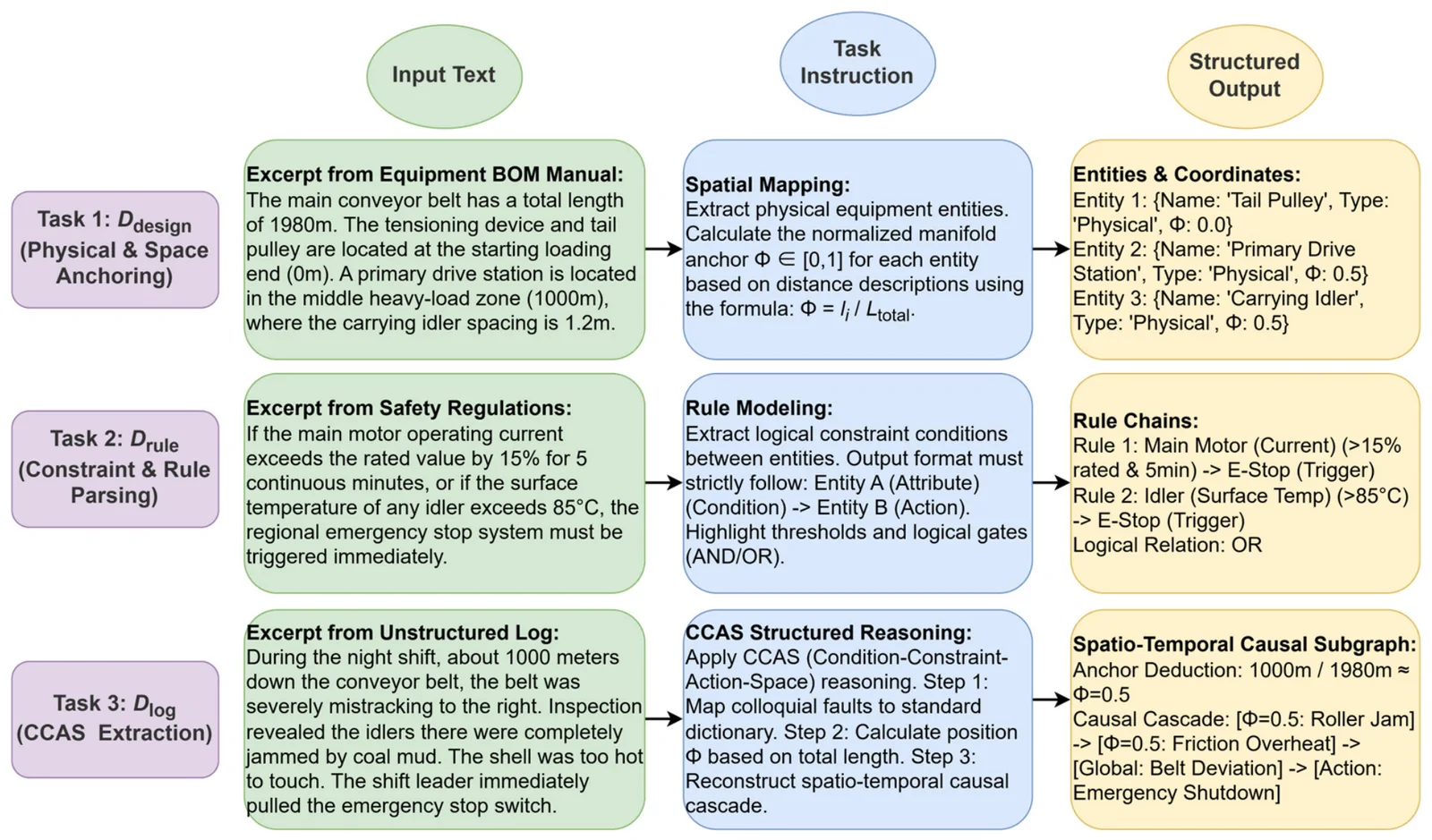
Parallel extraction architecture for heterogeneous data.

Figure 3 illustrates the parallel knowledge extraction architecture for heterogeneous data sources. 

According to the structural regularity of the input text, three extraction pipelines are assigned: Task 1 computes static physical anchors from engineering specifications using spatial mapping formulas; Task 2 constructs rigid rule chains from state-triggered regulatory descriptions; and Task 3 applies CCAS reasoning to unstructured inspection logs for dynamic spatiotemporal parsing. In Task 3, the extraction instructions guide state-dictionary alignment and spatial arithmetic inference, transforming non-standard colloquial records into causal subgraphs containing an absolute coordinate, multiple fault nodes, and their corresponding maintenance actions. This process supports the extraction of implicit temporal and spatial information from unstructured maintenance records.

### 3.4. Linear Topology-Aware Alignment Algorithm

Due to the structural homogeneity of linear assets, entity alignment algorithms based solely on semantic similarity tend to incorrectly merge identically named components located at different positions. The proposed LTA-Alignment algorithm addresses this issue by introducing a spatial penalty term and a logical arbitration mechanism. First, for a candidate entity pair (*e_i_*,*e_j_*), the textual descriptions of the two entities are encoded using the Qwen/Qwen3-Embedding-0.6B checkpoint corresponding to the Hugging Face snapshot dated 16 June 2025 (revision b92a382). The model generates 1024-dimensional semantic representations **v***_i_* and **v***_j_* using its default last-token pooling strategy, followed by L2 normalization. The semantic similarity between the two candidate entities is then calculated using cosine similarity [33]:

(12) $${Sim}_{sem}\left(e_{i},e_{j}\right)=\frac{v_{i}\cdot v_{j}}{\left\|v_{i}\right\|\left\|v_{j}\right\|}$$

Absolute deviation *δ* between two entities in the manifold space is calculated:

(13) $$\delta \left(e_{i},e_{j}\right)=\left|\Phi \left(e_{i}\right)-\Phi \left(e_{j}\right)\right|$$

A Gaussian kernel function is introduced to construct the spatial topological penalty term *Sim_top_* [34], where *σ* is a hyperparameter controlling spatial tolerance:

(14) $${Sim}_{top}\left(e_{i},e_{j}\right)=exp\left(-\frac{\delta {\left(e_{i},e_{j}\right)}^{2}}{2\sigma^{2}}\right)$$

The final alignment score *S_align_* is defined as the product of semantic similarity and the topological penalty term:

(15) $$S_{align}\left(e_{i},e_{j}\right)={Sim}_{sem}\left(e_{i},e_{j}\right)\cdot {Sim}_{top}\left(e_{i},e_{j}\right)$$

To balance computational cost and alignment accuracy, a dual-threshold mechanism (*τ_low_*,*τ_high_*) is introduced to establish a piecewise arbitration strategy. The decision output D is defined as follows. When the score falls within an ambiguous interval, an LLM-based arbitrator F_LLM_ is triggered, and historical context *C_ctx_* is incorporated for logical verification:

(16) $$D\left(e_{i},e_{j}\right)=\{\begin{array}{c} \mathrm{Reject},\;S_{align}<\tau_{low}\\ \mathrm{Merge},\;S_{align}>\tau_{high}\\ F_{LLM}\left(e_{i},e_{j},C_{ctx}\right),\;\tau_{low}\leq S_{align}\leq \tau_{high} \end{array}$$

Algorithm 1 presents the complete pseudocode execution logic of Linear Topology-Aware Alignment (LTA-Alignment).
**Algorithm 1** LTA-Alignment with LLM Arbiter**Input:** Candidate entity pair (*e_i_*,*e_j_*), Total linear length *L_total_*, Semantic threshold [*τ_low_*,*τ_high_*], Gaussian tolerance *σ*. **Output:** Alignment decision *D*∈{*Merge*,*Reject*}  1: **Phase 1: Gaussian Topological Penalty Computation** 2: Extract semantic embeddings **v***_i_*,**v***_j_* for *e_i_*,*e_j_* 3: *Sim_sem_* ← *CosineSimilarity*(**v***_i_*,**v***_j_*)  4: Extract absolute physical distances *l_i_*,*l_j_* for *e_i_*,*e_j_* 5: $\mathrm{Compute}\;\mathrm{normalized}\;\mathrm{manifold}\;\mathrm{anchors}:\;\Phi \left(e_{i}\right)\leftarrow \frac{l_{i}}{L_{total}},\Phi \left(e_{j}\right)\leftarrow \frac{l_{j}}{L_{total}}$ 6: $\mathrm{Compute}\;\mathrm{absolute}\;\mathrm{spatial}\;\mathrm{deviation}:\;\delta \leftarrow \left|\Phi \left(e_{i}\right)-\Phi \left(e_{j}\right)\right|$ 7: $\mathrm{Compute}\;\mathrm{topological}\;\mathrm{penalty}\;\mathrm{multiplier}:\;Sim_{top}\leftarrow exp\left(-\frac{\delta^{2}}{2\sigma^{2}}\right)$ 8: $\mathrm{Compute}\;\mathrm{composite}\;\mathrm{alignment}\;\mathrm{score}:\;S\leftarrow Sim_{sem}\times Sim_{top}$
 9: **Phase 2: Grey-Zone Arbitration Logic** 10: **if** *S* < *τ_low_* **then** 11:      D←Reject (Short-circuit rejection)  12: **else if** *S* > *τ_high_* **then** 13:      D←Merge (Direct unconditional merge) 14: **else** (Grey-Zone triggered) 15:      *C_ctx_* ← *ExtractContext*(*e_i_*,*e_j_*) (e.g., timestamps, worker logs) 16:      *Pprompt ← ConstructPrompt(e_i_,e_j_,C_ctx_)* 17:      *decision_result ← LLM_Arbiter(P_prompt_)*
 18:      **if** *decision_result* == “*Yes*” **then** 19:          D←Mege 20:      **else** 21:          D←Reject 22:          **end if** 23: **end if** 24: **return:** D

The pseudocode clearly illustrates how the spatial penalty term rapidly attenuates the overall alignment score of identically named components at different locations, and specifies the trigger conditions for invoking the large language model when the score falls within the ambiguous interval.

### 3.5. Dynamic Evolution and Updating of the Spatiotemporal Knowledge Graph

The health state of industrial equipment degrades continuously over time. The knowledge graph must support dynamic evolution. Let the graph state at time step *t* be denoted as $G_{t}$. The iterative update can be formulated as a set operation over incremental and obsolete graph elements:

(17) $$G_{t+1}=\left(G_{t}\cup \Delta G_{new}\right)-\Delta G_{obs}$$

where $\Delta G_{new}$ denotes the newly extracted spatiotemporal relations generated by the CCAS mechanism, and $\Delta G_{obs}$ denotes obsolete rules or resolved faults.

Considering the timeliness of maintenance logs, an exponential decay confidence function *C*(*e*,*t*) is introduced for newly constructed fault-related entities:

*C*(*e*,*t*) = *C*_0_∙*exp*(−*λ*(*t* − *t*_0_)) (18)

where *C*_0_ denotes the initial confidence of a newly activated fault-related entity, *t*_0_ denotes its occurrence time, and *λ* controls the temporal decay rate. The elapsed time *t* − *t*_0_ is measured in days. An archival threshold *C*_arch_ is introduced to distinguish low-confidence historical fault states. Fault-related entities satisfying *C*(*e*,*t*) < *C*_arch_ are transferred to the historical state and excluded from current-priority reasoning.

## 4. Experiments and Evaluation

The physical span of long-distance linear assets is extremely large. Full-scale fault tracing based entirely on distributed sensors in real environments incurs prohibitive costs. Therefore, the evaluation focuses on algorithmic performance and on the capability of large language models to reconstruct logical structures from highly noisy text. The validation process integrates real historical operation and maintenance data with algorithmic augmentation.

### 4.1. Construction of a Multi-Source Real and Augmented Industrial Text Dataset

Belt conveyor systems exhibit strong characteristics of highly duplicated component names and extreme spatial dispersion in industrial scenarios. To rigorously evaluate the capability of the proposed framework in handling long-distance linear topology, a large-scale spatiotemporal maintenance text dataset is constructed based on real operational records from a mining enterprise, combined with algorithmic augmentation. The physical entity repository is directly derived from the engineering bill of materials of real equipment. The system includes 11,850 component entities and their associated descriptive texts. To simulate realistic alignment conflicts, the dataset preserves and amplifies the phenomenon of identical naming. For a system with a belt width of 1.6 m and a total length of 1980 m, the dataset includes 1650 sets of carrying trough rollers and 660 sets of return flat rollers. These 2310 components share highly similar textual descriptions. Each component is assigned a unique absolute spatial scalar mapped onto a 1980-m physical manifold. Real inspection and repair records collected from field operators were used as the core maintenance-log data. The original records were written in the local language. A domain-specific language model generated the initial English translations. Domain annotators subsequently checked component names, fault states, maintenance actions, causal order, and spatial expressions against the original records. The verified English corpus was then used consistently for gold-standard annotation, prompt-based extraction, entity alignment, and baseline comparison.

To further evaluate robustness under complex industrial text conditions, the dataset is expanded through rule-guided text augmentation based on standardized real maintenance logs. The augmentation process uses manually checked real logs as seed samples. The original fault type, component category, spatial location, and maintenance action are preserved, while only the textual expression is perturbed and extended. Three operations are applied: introducing common field abbreviations, colloquial expressions, and non-standard state descriptions; combining adjacent fault states to generate composite fault narratives with temporal order and spatial variation; and rewriting inspection findings, handling actions, and risk descriptions without changing the root-cause node or spatial anchor. A total of 3500 text samples are constructed for system evaluation. Among the 3500 log entries, 875 entries were anonymized real seed logs, and 2625 entries were rule-guided augmented variants derived from the same 875 original maintenance events. The augmented variants were used only to increase linguistic diversity, including field abbreviations, colloquial expressions, non-standard fault descriptions, and composite fault narratives. The original fault type, component identity, spatial anchor, root-cause node, and maintenance action were kept unchanged during augmentation. The entity labels, spatial anchors, and causal-relation labels of augmented samples are inherited from the corresponding seed samples and further checked by rule-based validation and manual terminology review. In addition, the dataset includes 8230 gold-standard knowledge triples constructed through the structured annotation protocol for subsequent extraction and alignment evaluation. Table 1 presents the detailed statistics of the constructed dataset.

To ensure experimental traceability, the dataset composition, data partitioning, model-input boundary, and parameter settings are uniformly controlled. The experimental data consist of three parts: physical entity data derived from real belt conveyor bills of materials and structural records; maintenance-log data collected from field inspection, repair, and fault-handling records after anonymization and standardization; and gold-standard annotation data containing entity types, fault states, spatial anchors, causal relations, and maintenance actions. In this study, gold-standard annotations refer to reference labels constructed from objective engineering evidence and verified through the structured annotation protocol.

To avoid augmentation-induced information leakage, the dataset was split at the seed-log/event level. Each real seed log and its three augmented variants were assigned together to the same subset. Specifically, the training set contained 612 real seed logs and 1836 augmented logs, the validation set contained 88 real seed logs and 264 augmented logs, and the test set contained 175 real seed logs and 525 augmented logs. The final training, validation, and test sets therefore contained 2448, 352, and 700 log entries, respectively, corresponding to an approximately 7:1:2 partition ratio. To prevent the larger augmented subset from dominating the reported performance, all test metrics were additionally calculated for the 175 real seed logs and the 525 rule-guided augmented logs separately. The results obtained from the 700-log combined test set were retained as aggregate references. The real-log subset was used to assess performance on directly collected industrial records, whereas the augmented subset was used to evaluate robustness to controlled linguistic perturbations. Each test event consisted of one real seed log and three rule-guided variants. Sample-level metrics were calculated separately for the real, augmented, and combined subsets. Statistical inference used the underlying maintenance event as the clustering unit, with all four records derived from the same event retained within the same cluster. No threshold, prompt, or model parameter was adjusted using either test subset. The training set is used for prompt design and rule induction. The validation set is used to determine the thresholds and spatial tolerance of LTA-Alignment and to select the temporal decay constant *λ* of Dynamic STKG. The test set is used only for final evaluation and is not involved in prompt construction, parameter selection, or output correction. To prevent test information leakage, the gold-standard root-cause nodes, standardized spatial anchors, and entity-merge labels in the test set are not provided to the large language model. For each log, the model input includes only the original text, equipment topology rules, spatial conversion formulas, and output-format constraints. The knowledge extraction results are automatically generated under the CCAS schema and compared with the gold-standard annotations. Entity alignment is determined by semantic similarity, normalized spatial distance, and Gaussian topological penalty. Fault traceability retrieves root-cause nodes only from the alarm phenomenon, historical maintenance records, and spatial descriptions.

**Figure 4:**
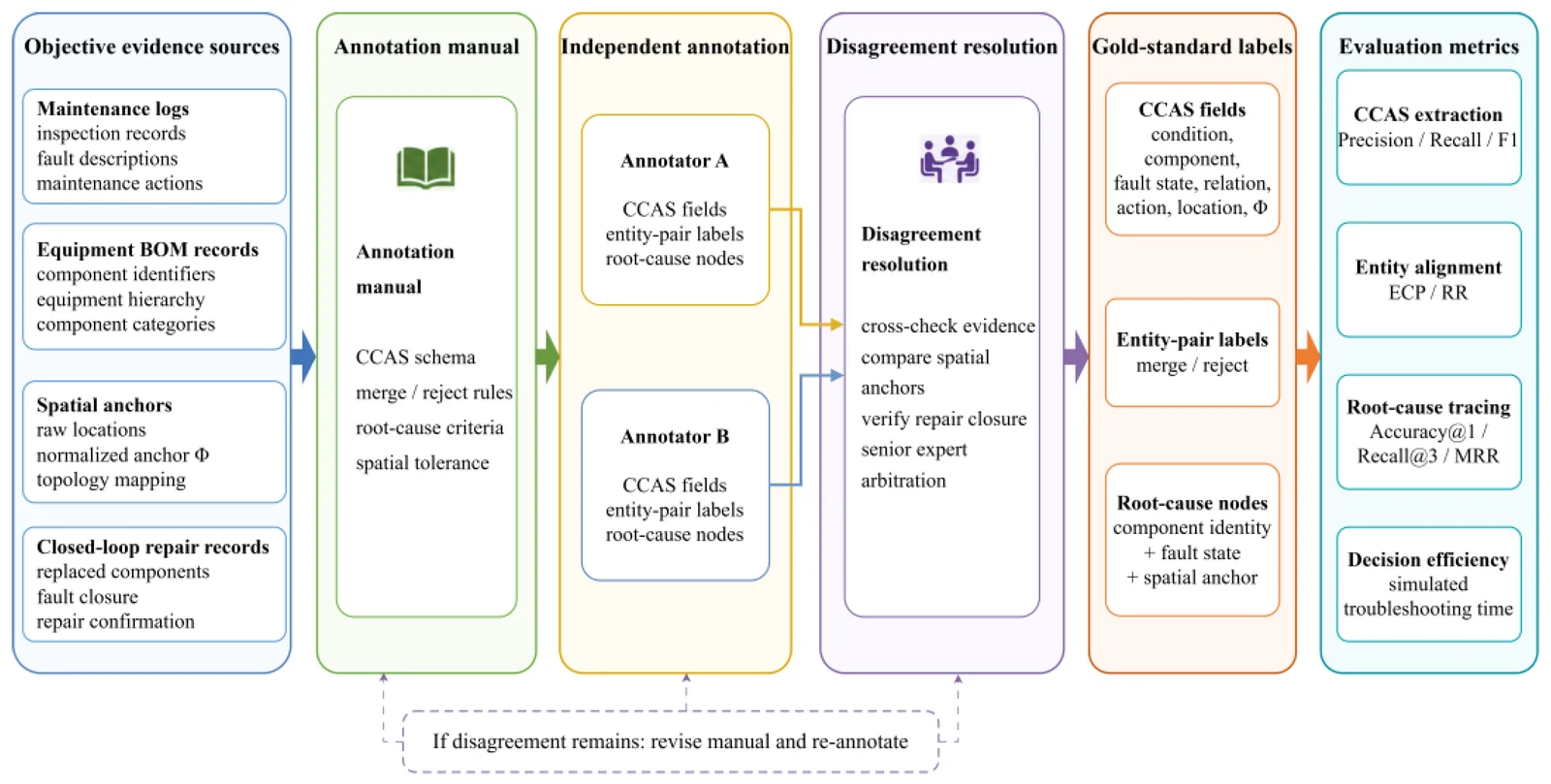
Gold-standard annotation and evaluation protocol.

To construct reliable gold-standard annotations, a structured annotation protocol was adopted instead of relying on direct expert judgment alone. Initial labels were derived from four types of evidence: anonymized maintenance logs, equipment BOM records, normalized spatial anchors, and closed-loop repair records. The annotation targets included CCAS fields, entity merge/reject labels, and root-cause nodes for downstream traceability. Two annotators with mining equipment maintenance backgrounds independently annotated each sample according to a predefined annotation manual. Disagreements were resolved through cross-checking of equipment identity, spatial anchor, maintenance context, and repair closure evidence. Ambiguous cases that could not be supported by at least one objective evidence source were excluded from the final evaluation set or submitted to a senior domain expert for final arbitration. To further clarify how gold-standard labels were constructed and linked to subsequent evaluation metrics, the complete annotation and evaluation protocol is shown in Figure 4.

As shown in Figure 4, the protocol starts from four objective evidence sources, including anonymized maintenance logs, equipment BOM records, normalized spatial anchors, and closed-loop repair records. These evidence sources are first organized according to a predefined annotation manual, which specifies the CCAS schema, entity merge/reject rules, root-cause matching criteria, and spatial tolerance. Two annotators then independently generate CCAS fields, entity-pair labels, and root-cause nodes. When inconsistencies occur, the labels are determined through evidence cross-checking and senior expert arbitration. The resulting gold-standard labels are finally used for field-level F1 evaluation, *ECP*/*RR* calculation, root-cause ranking metrics, and simulated troubleshooting time estimation. This protocol ensures that expert judgment is used mainly for ambiguity resolution rather than direct label generation.

Qwen/Qwen2.5-14B-Instruct-AWQ, corresponding to the Hugging Face snapshot dated 28 April 2025 (revision 1cae09f), was used for CCAS-based knowledge extraction and grey-zone arbitration. Qwen/Qwen3-Embedding-0.6B, corresponding to the snapshot dated 16 June 2025 (revision b92a382), was used to generate 1024-dimensional entity embeddings for semantic similarity calculation in LTA-Alignment. For each model, the checkpoint and tokenizer were loaded from the same fixed snapshot. Both models were deployed locally. The instruction-tuned language model was served through vLLM with AWQ 4-bit quantization, and the embedding model was loaded through Sentence Transformers. Dify v1.6.0 was used for workflow orchestration and system demonstration, whereas all quantitative experiments were executed using Python 3.10 scripts. The checkpoint and tokenizer revisions, inference-library versions, prompt templates, decoding parameters, input order, batch size, and software environment were kept fixed throughout the experiments. Three independent LLM inference runs were conducted using three predefined fixed random seeds. For each run, all test logs and all candidate pairs entering the grey zone were processed independently under the same configuration. Run-level predictions were retained and evaluated separately for all mean ± standard-deviation comparisons. After run-level evaluation, two-of-three majority voting was applied to the discrete CCAS fields and to the grey-zone MERGE/REJECT decisions. The normalized spatial anchor was aggregated using the median of the three valid numerical outputs. The aggregated outputs were used to construct the final structured records and knowledge graph, calculate aggregate grey-zone arbitration accuracy, and support visualization and qualitative case analysis. The embedding model and deterministic alignment settings used identical fixed inputs across the three runs. The main experimental parameters are listed in Table 2.

The main comparative results are reported as the mean ± sample standard deviation over three independent runs. Trainable baselines were independently trained or fine-tuned using three fixed training seeds, while the LLM-based modules were independently executed using the corresponding inference seeds. Deterministic methods operating on identical fixed inputs produced zero run-to-run variance. Paired cluster-bootstrap tests with 10,000 resamples were used for Global F1, entity-alignment correctness, and Accuracy@1. Resampling was performed at the underlying-event level, with records and candidate pairs associated with the same seed event sampled together. All tests were two-sided at a significance level of 0.05, with Holm correction applied to the key comparisons.

To clarify how gold-standard labels were constructed and used for evaluation, Table 3 presents representative annotation examples for different evaluation tasks. The examples cover CCAS extraction, entity alignment, entity rejection, and downstream root-cause tracing. For confidentiality, enterprise names, personnel information, real equipment identifiers, and production dates were removed or anonymized, while fault states, maintenance actions, spatial descriptions, and evidence links were retained. These examples show that the evaluation labels were constructed from structured evidence and predefined matching criteria rather than from direct subjective judgment.

To prevent uncontrolled generation and format drift during industrial text extraction, a strongly constrained prompt template is adopted. The output format is strictly restricted to JSON structures. The core prompt template is presented in Table 4. During inference, each input comprised the original maintenance-log text, equipment-topology rules, spatial-conversion formulas, and output-format constraints. Gold-standard test annotations were introduced during the subsequent evaluation stage. The resulting JSON records contained the extracted CCAS fields and grey-zone arbitration decisions used for metric calculation.

Before using the combined test set for subsequent model comparisons, CCAS extraction and LTA-Alignment were evaluated separately on the 175 real seed logs and the 525 rule-guided augmented logs. The two subsets used identical model checkpoints, prompt templates, alignment thresholds, spatial tolerance, and evaluation criteria. Each subset was evaluated independently under the three predefined runs. Table 5 reports the run-averaged results for the real, augmented, and combined subsets.

As shown in Table 5, the global F1-score decreased from 95.00% on the real seed logs to 93.84% on the augmented logs. The decrease is consistent with the augmentation process, which introduces abbreviations, colloquial expressions, non-standard fault descriptions, composite narratives, and alternative spatial expressions. The combined F1-score of 94.10% falls between the two subset-level results and is closer to the augmented result because augmented logs account for 75% of the test set. For entity alignment, *ECP* remained above 96% across all three subsets. The small increase in *RR* on the augmented and combined subsets occurred together with stable cluster purity, indicating that the additional compression mainly reflected valid merging of paraphrastic records. The combined test set was retained as an aggregate benchmark, while the real and augmented subsets characterize performance on field records and controlled linguistic variants, respectively.

The empirical scope of the dataset comprises one enterprise, one 1980 m non-branching conveyor topology, and a verified English maintenance-log corpus. Deployment on another conveyor line requires line-specific BOM mappings, equipment dictionaries, topology rules, spatial-expression normalization, and validation-based calibration of *τ_low_*, *τ_high_*, and *σ*. Cross-enterprise transfer, branching conveyor networks, and direct processing of original-language logs require further validation.

### 4.2. Robustness and Performance Evaluation of Knowledge Extraction

The core objective of this experiment is to compare the proposed framework, driven by large language model reasoning, with conventional models in terms of their intrinsic differences in extracting implicit knowledge triplets from highly noisy industrial maintenance logs. To ensure a comprehensive evaluation, representative models from three developmental stages of information extraction are selected as baselines. These include the classical sequence labeling framework BiLSTM-CRF based on bidirectional long short-term memory and conditional random fields [35], the deep pre-trained model BERT-base that captures semantic features via a masking mechanism [36], and the sequence-to-sequence generative architecture T5 with strong generalization ability for structured text [37]. For CCAS evaluation, all models were tested on the same held-out test set under identical input conditions. Precision, Recall, and F1-score were calculated at the structured-item level by comparing model outputs with the gold-standard annotations. The evaluated items included condition, component, fault state, causal relation, maintenance action, raw location, and normalized spatial anchor. A spatial anchor was counted as correct only when its normalized coordinate error was not larger than 0.02. The reported global F1-score was calculated as the macro-level average over all structured extraction items rather than as a subjective overall judgment. The evaluation metrics are defined as follows:

Precision = *TP*/(*TP* + *FP*) (19)

Recall = *TP*/(*TP* + *FN*) (20)

F1 = 2 × Precision × Recall/(Precision + Recall)(21)

F1_*global*_ = (1/*M*) × ∑_*i*=1_^*M*^ F1_*i*_ (22)

where *TP*, *FP*, and *FN* denote the number of true-positive, false-positive, and false-negative structured items, respectively. *M* denotes the number of structured evaluation fields. In this study, *M* = 7, corresponding to condition, component, fault state, causal relation, maintenance action, raw location, and normalized spatial anchor. The field-level evaluation units and matching criteria are summarized in Table 6.

**Figure 5:**
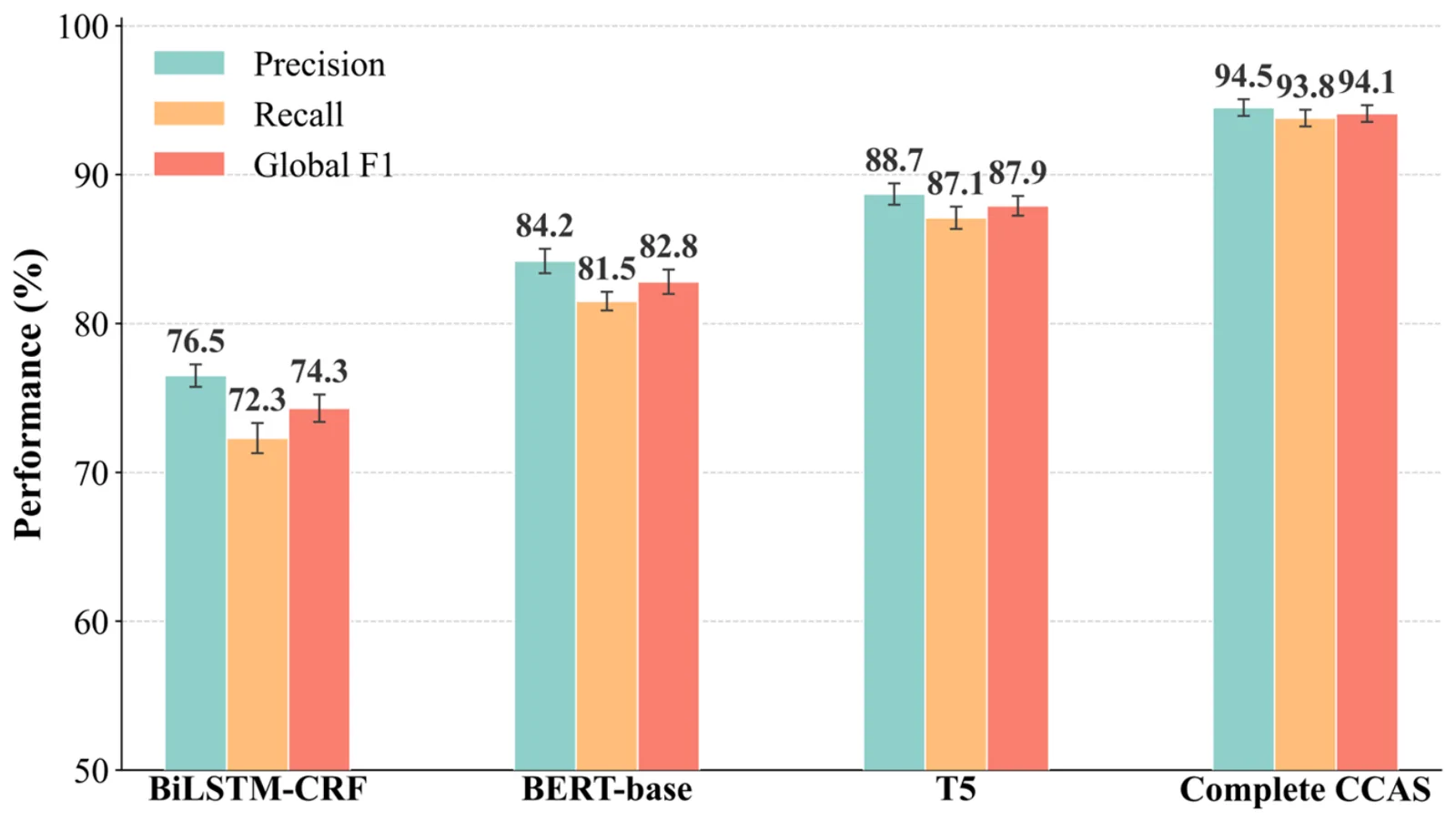
Performance comparison of different knowledge extraction models.

Based on the above field-level evaluation protocol, the quantitative results of the four knowledge extraction models on the noisy industrial log dataset are shown in Figure 5.

The bars present the mean values over three independent runs, and the error bars denote one sample standard deviation. BiLSTM-CRF and BERT-base achieved Global F1 scores of 74.30 ± 0.92% and 82.80 ± 0.82%, respectively. Their lower recall indicates that token-level sequence modeling remains sensitive to colloquial expressions and irregular entity boundaries. T5 improved the Global F1 score to 87.90 ± 0.66% through generative sequence modeling, but its performance remained limited when implicit causal and spatial relations had to be reconstructed. Complete CCAS achieved a Precision of 94.50 ± 0.56%, a Recall of 93.80 ± 0.55%, and a Global F1 score of 94.10 ± 0.56%. The 6.20-percentage-point improvement in Global F1 over T5 remained significant under the event-cluster-aware paired bootstrap after Holm correction (*p_adj_* = 0.004). The proposed scheme uses contextual reasoning and engineering knowledge to complete missing causal descriptions and then constrains the generated content through the CCAS structured output format. This combination supports consistent extraction of fault states, causal relations, maintenance actions, raw locations, and normalized spatial anchors from noisy maintenance logs. BiLSTM-CRF, BERT-base, and T5 were independently trained and evaluated three times under the same data partition.

**Figure 6:**
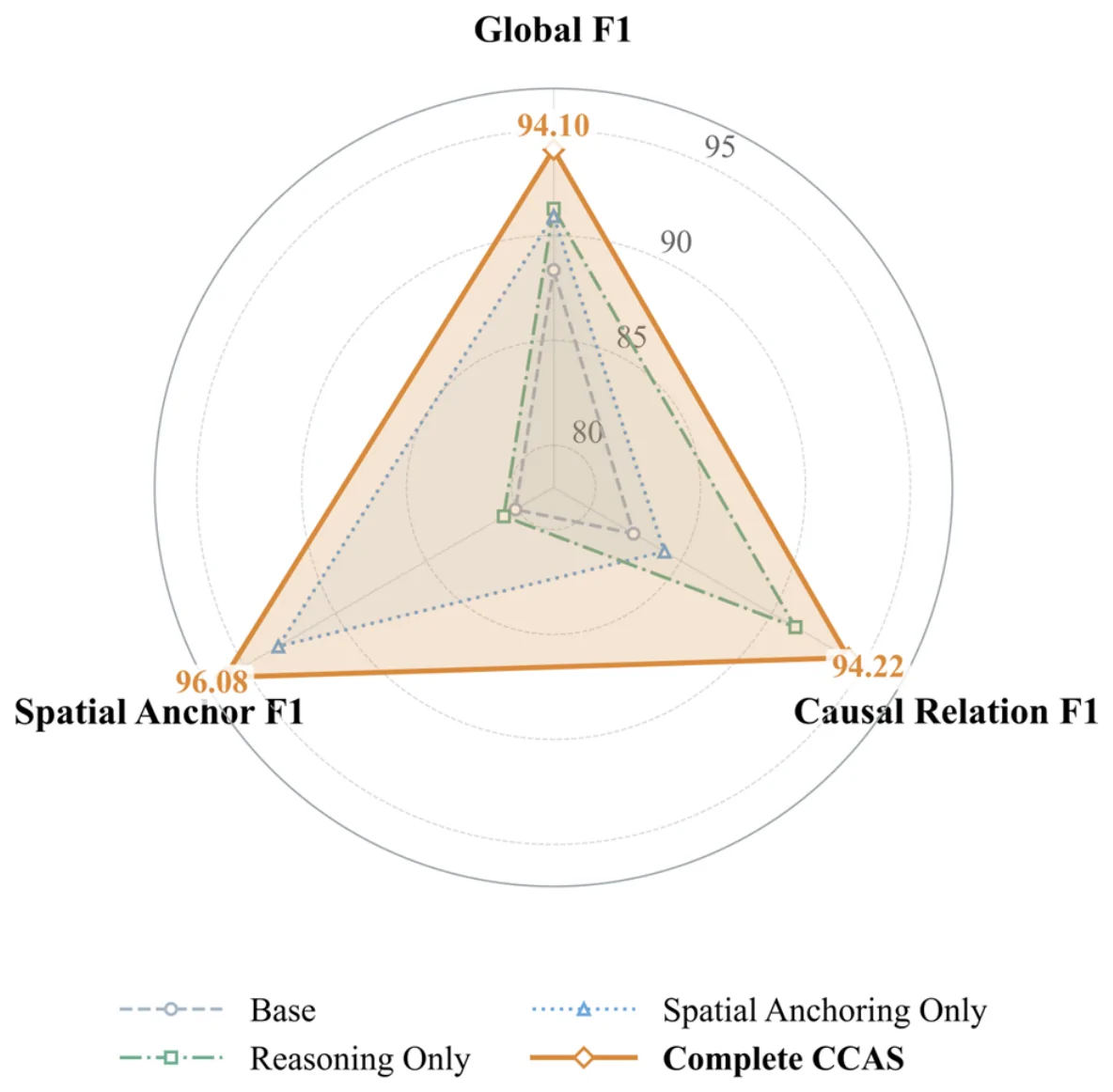
Radar visualization of the factorial ablation results for step-by-step reasoning and spatially anchored prompting in CCAS extraction.

To further isolate the contributions of step-by-step structured reasoning and spatially anchored prompting in CCAS extraction, four prompt configurations were evaluated on the same 700-log test set. All configurations used the same Qwen/Qwen2.5-14B-Instruct-AWQ checkpoint, data partition, JSON output schema, decoding parameters, and three predefined inference runs. The base configuration retained the constrained JSON output format but removed both staged reasoning instructions and explicit spatial-normalization rules. The reasoning-only configuration introduced step-by-step extraction of abnormal conditions, physical components, causal relations, and maintenance actions. The spatial-anchoring-only configuration introduced topology rules and explicit normalization of location expressions using Φ = *l*/*L_total_*. The complete CCAS configuration combined both components. The ablation results are visualized in Figure 6.

As shown in Figure 6, the base constrained prompt achieved a Global F1-score of 88.36%, a causal relation F1-score of 82.40%, and a spatial anchor F1-score of 80.10%. Introducing step-by-step reasoning increased the Global F1-score to 91.28% and the causal relation F1-score to 91.30%, while the spatial anchor F1-score changed only marginally to 80.74%. Introducing spatial anchoring increased the Global F1-score to 90.93% and the spatial anchor F1-score to 93.14%, while the causal relation F1-score increased modestly to 84.10%. The complete CCAS configuration achieved the best overall performance, with a Global F1-score of 94.10%, a causal relation F1-score of 94.22%, and a spatial anchor F1-score of 96.08%. Relative to the base configuration, the complete CCAS improved Global F1 by 5.74 percentage points, indicating that step-by-step reasoning and topology-constrained spatial inference provide complementary benefits for industrial maintenance-text extraction.

### 4.3. Comparative and Ablation Study of the LTA-Alignment Mechanism

To evaluate the proposed LTA-Alignment method, an external comparison and a controlled ablation study were conducted. SCSA [23] was selected as the external entity-alignment method. The engineering BOM and equipment-topology data were organized as the source knowledge graph, while the entities, attributes, and relations extracted from maintenance logs formed the target knowledge graph. Training-set correspondences were used as alignment seeds for SCSA. The three LTA-Alignment parameters, *σ*, *τ_low_*, and *τ_high_*, were jointly selected exclusively on the validation set before the test set was accessed. The ablation study included three progressive settings. Setting A performs alignment using cosine similarity between normalized semantic embeddings. Setting B incorporates the Gaussian spatial compatibility term. Setting C further introduces LLM-based grey-zone arbitration. After all parameters were fixed, the methods were evaluated on the same independent test set containing 12,600 candidate entity pairs, including 3780 positive MERGE pairs and 8820 negative REJECT pairs. Each candidate pair retained the seed-event identifier associated with its log-derived entity, which was subsequently used to group related pairs during statistical resampling. Merge Precision, Merge Recall, and Merge F1 were calculated at the candidate-pair level. Entity Cluster Purity (*ECP*) [38] and Reduction Rate (*RR*) [39] were calculated from the resulting entity clusters.

The *RR* measures the overall compression ratio of initial isolated nodes after alignment. It is defined as:

(23) $$RR=\frac{\left|E_{init}\right|-\left|E_{align}\right|}{\left|E_{init}\right|}\times 100\%$$

where |*E_init_*| denotes the total number of initial entities from multi-source inputs before alignment, and |*E_align_*| denotes the number of remaining entities after fusion and disambiguation. *RR* quantifies the degree of graph compression and is interpreted jointly with *ECP* and pairwise Merge Precision, Recall, and F1. An increase in *RR* accompanied by stable or improved cluster purity and pairwise alignment quality indicates effective removal of redundant entities. An increase in *RR* accompanied by declining *ECP* or Merge Precision indicates over-merging, while a low *RR* together with limited Merge Recall indicates conservative alignment and residual graph fragmentation. The desirable operating regime therefore provides increased graph compression while maintaining entity-level and cluster-level alignment quality. Entity Cluster Purity (*ECP*) evaluates the physical consistency of the complete entity partition produced after alignment. Following the standard definition in clustering analysis, it is computed as:

(24) $$ECP=\frac{1}{\left|E_{init}\right|}\sum_{i=1}^{K}\underset{j}{\max}\left|C_{i}\cap G_{j}\right|$$

where *C_i_* denotes the *i*-th output entity cluster after alignment, including both multi-entity clusters and singleton clusters, and *K* denotes the total number of output clusters. The output clusters form a complete and mutually disjoint partition of *E_init_*, such that $\sum_{i=1}^{K}\left|C_{i}\right|=\left|E_{init}\right|.$ *G_j_* denotes the *j*-th gold-standard physical-entity equivalence class. The denominator |*E_init_*| therefore normalizes correctly assigned cluster memberships over all initial entities. A higher *ECP* indicates greater physical consistency of the output entity clusters [40]. *ECP* is interpreted jointly with *RR* and pairwise Merge Precision, Recall, and F1.

A joint grid search over *σ*, *τ_low_*, and *τ_high_* was conducted exclusively on the validation set. The value of σ ranged from 0.005 to 0.050, with denser sampling around the expected spatial-tolerance region. The value of *τ_low_* ranged from 0.45 to 0.65, and *τ_high_* ranged from 0.65 to 0.85, both at intervals of 0.05 and subject to *τ_low_* < *τ_high_*. For parameter selection, macro-F1 was defined as the arithmetic mean of the class-level F1-scores for the MERGE and REJECT classes. The maximum validation macro-F1 was 96.49%. Among the parameter combinations whose macro-F1 was within 0.10 percentage points of this maximum, (*σ*,*τ_low_*,*τ_high_*) = (0.02,0.55,0.75) produced the lowest grey-zone coverage, achieving a validation macro-F1 of 96.42% and grey-zone coverage of 17.86%. All three parameters were then fixed before test-set evaluation. Section 4.4 subsequently provides a post-hoc sensitivity analysis of *ECP* and *RR* around the validation-selected value of *σ*, without further parameter selection. Decision-level majority voting was subsequently applied to the 2284 unique grey-zone pairs. The final output contained 906 true-MERGE, 1253 true-REJECT, 51 false-MERGE, and 74 false-REJECT decisions, yielding an arbitration accuracy of 94.53%. Within the grey zone, Merge Precision, Recall, and F1 were 94.67%, 92.45%, and 93.55%, respectively. The false-MERGE rate was 3.91% among the 1304 gold-standard REJECT pairs, while the false-REJECT rate was 7.55% among the 980 gold-standard MERGE pairs. False-MERGE decisions reduce physical cluster purity, whereas false-REJECT decisions retain redundant entities and limit graph reduction. Based on locally recorded vLLM usage and latency, one complete arbitration run consumed approximately 1.14 million tokens. The mean end-to-end inference latency was 1.64 ± 0.37 s per pair, corresponding to 62.43 min of serial execution.

**Figure 7:**
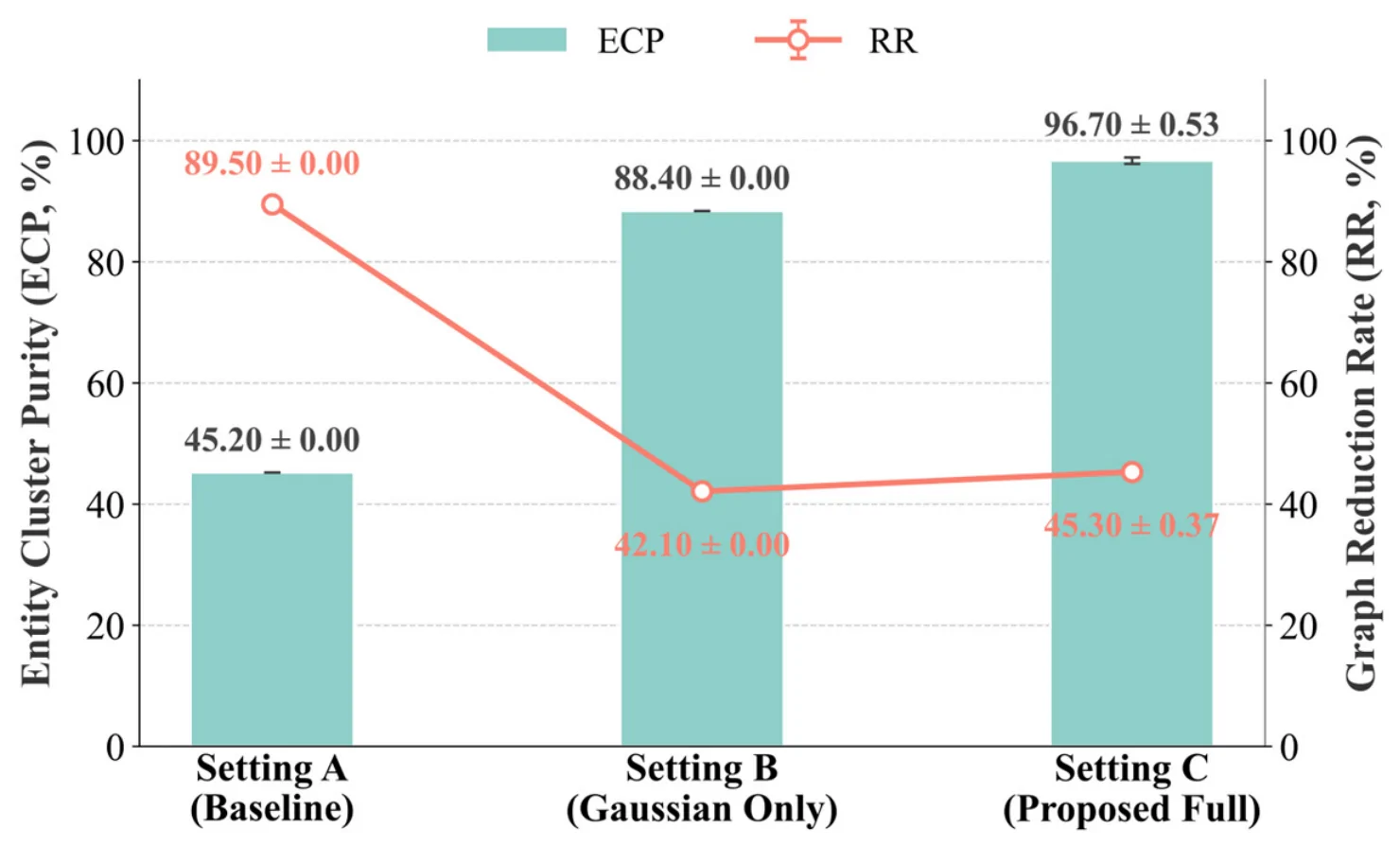
Dual-axis comparison of core metrics in the ablation study of the LTA-Alignment algorithm.

The quantitative results of the ablation study are shown in Figure 7. Settings A and B operate on fixed candidate pairs, semantic embeddings, spatial anchors, thresholds, and Gaussian parameters. Their repeated evaluations therefore produced identical outputs. Setting A, which relies only on semantic similarity, achieved an *ECP* of 45.20 ± 0.00% and an *RR* of 89.50 ± 0.00%. The combination of high graph compression and low cluster purity indicates extensive merging of entities belonging to different physical components. After the Gaussian topological penalty was introduced, Setting B increased *ECP* to 88.40 ± 0.00% and reduced *RR* to 42.10 ± 0.00%. This joint change shows that the spatial constraint suppressed a large proportion of invalid cross-location merges and produced a more conservative compression regime.

Setting C further applies LLM-based arbitration to candidate pairs within the dual-threshold interval. In each run, the semantic similarities, Gaussian topological scores, candidate pairs, and decision thresholds were kept fixed, while all grey-zone pairs were independently re-arbitrated using the corresponding inference seed. The arbiter evaluated timestamps, shift records, maintenance context, and fault-evolution evidence. Setting C achieved an *ECP* of 96.70 ± 0.53% and an *RR* of 45.30 ± 0.37%. Compared with Setting B, mean *ECP* increased by 8.30 percentage points and mean *RR* increased by 3.20 percentage points. The simultaneous increases in *ECP* and *RR* show that grey-zone arbitration recovered additional valid merges, increased effective graph compression, and improved the physical consistency of the resulting entity clusters. Setting C achieved significantly higher pairwise alignment correctness than Setting B under the event-cluster-aware paired bootstrap, with candidate pairs grouped according to the seed events associated with their log-derived entities (*p_adj_* = 0.006 after Holm correction). The comparison with SCSA is presented in Table 7. SCSA was independently trained three times, while LTA-Alignment was evaluated using three independent grey-zone arbitration runs under the same data partition. Results are reported as the mean ± sample standard deviation.

As shown in Table 7, LTA-Alignment achieved mean Merge Precision, Recall, and F1 scores of 96.40%, 95.10%, and 95.75%, respectively, compared with 91.60%, 93.20%, and 92.39% for SCSA. Mean *ECP* increased from 92.80% to 96.70%. SCSA achieved a higher mean *RR* of 49.70%, while its Merge Precision, Merge Recall, Merge F1, and *ECP* were lower than those of LTA-Alignment. LTA-Alignment achieved an *RR* of 45.30% together with higher pairwise alignment quality and cluster purity, demonstrating a more favorable balance between graph compression and physical consistency.

**Figure 8:**
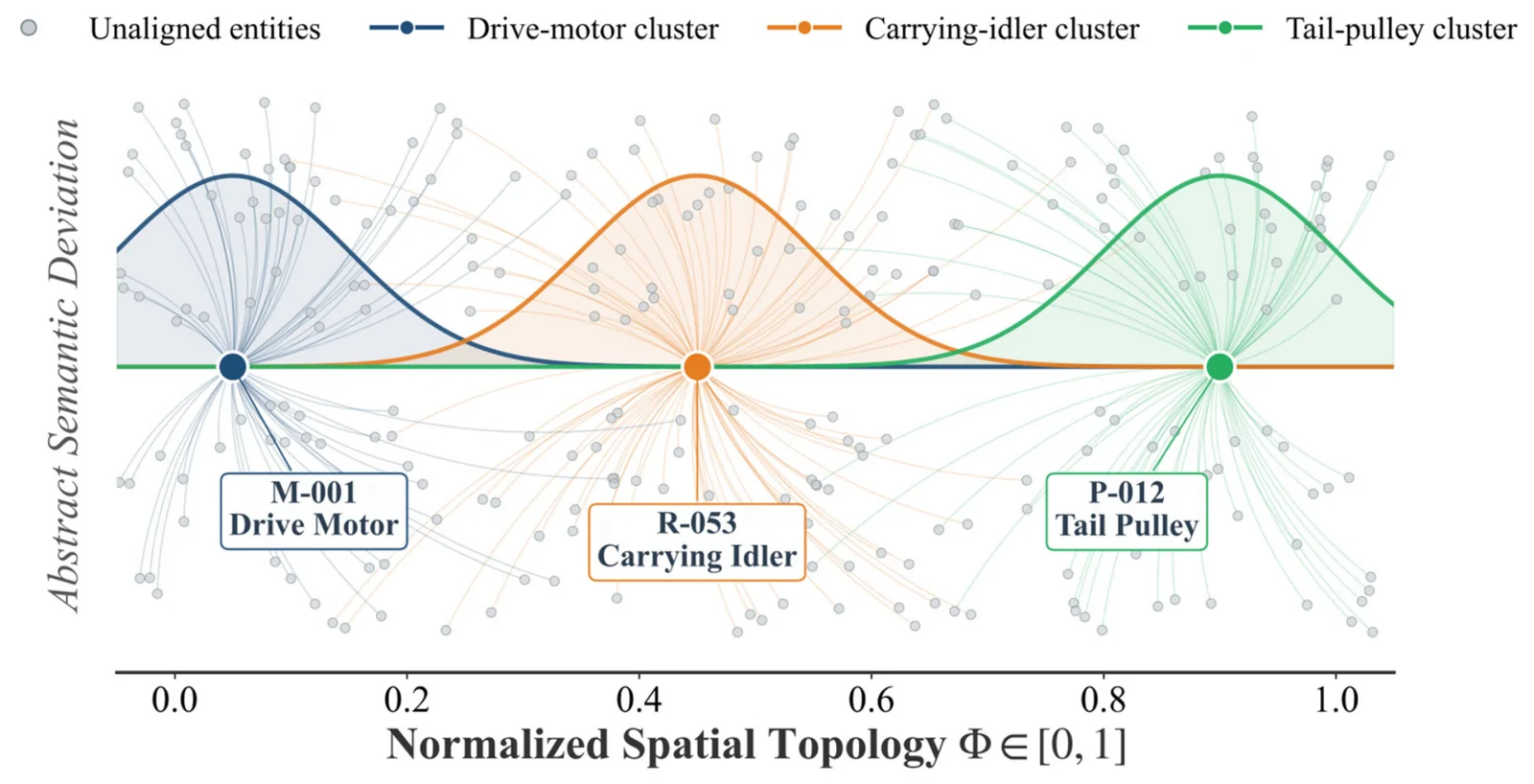
Visualization of entity aggregation and topological bias correction of the LTA-Alignment module in feature space.

To further reveal the physical meaning of the metric improvement from the feature space perspective, the spatial aggregation and alignment process is visualized, as shown in Figure 8. 

Gray dense scattered nodes represent chaotic semantic entities before alignment after multi-source ingestion. Overlapping Gaussian curves distributed along the normalized axis Φ ∈ [0, 1] form a strict mathematical penalty boundary. The visualization clearly demonstrates the attraction effect of the spatial topological field. The algorithm cuts off spurious similarity links between identically named nodes at different locations. Noisy isolated entities are guided to converge toward their true physical anchors, such as the carrying idler located at Φ = 0.45.

Table 8 presents a representative grey-zone entity pair with an alignment score of *S_align_* = 0.654. The two records exhibit high semantic similarity, with *S_sem_* = 0.980, whereas the normalized spatial deviation of δ = 0.018 reduces the Gaussian topological compatibility to *S_top_* = 0.667. Under Setting B, the reduced alignment score leaves the pair unmerged and produces a redundant entity node.

Setting C additionally incorporates component category, temporal order, fault-state evolution, and maintenance-action evidence. The abnormal-noise report recorded at 10:00 and the subsequent jamming diagnosis and bearing replacement recorded at 11:30 form a continuous maintenance-event sequence. The combined contextual evidence supports a MERGE decision under uncertainty in the reported spatial position. This case illustrates the contextual evidence used in grey-zone arbitration and is consistent with the aggregate arbitration performance reported above.

### 4.4. Parameter Sensitivity Analysis of Gaussian Topological Tolerance σ

After *σ* = 0.02 had been fixed through the joint validation search in Section 4.3, a post-hoc sensitivity analysis was conducted by varying *σ* over {0.005,0.010,0.015,0.020,0.025,0.030,0.040,0.050} on the validation set using a predefined fixed inference seed, while the remaining settings were held fixed.

The parameter *σ* controls the tolerance to spatial deviations in maintenance descriptions. A small value imposes an overly strict spatial constraint and may split descriptions of the same physical component into redundant nodes. In contrast, an excessively large value weakens the spatial penalty and increases the risk of merging identically named components at different locations. *ECP* and *RR* were jointly used to evaluate physical consistency and graph compression under different spatial-tolerance settings.

**Figure 9:**
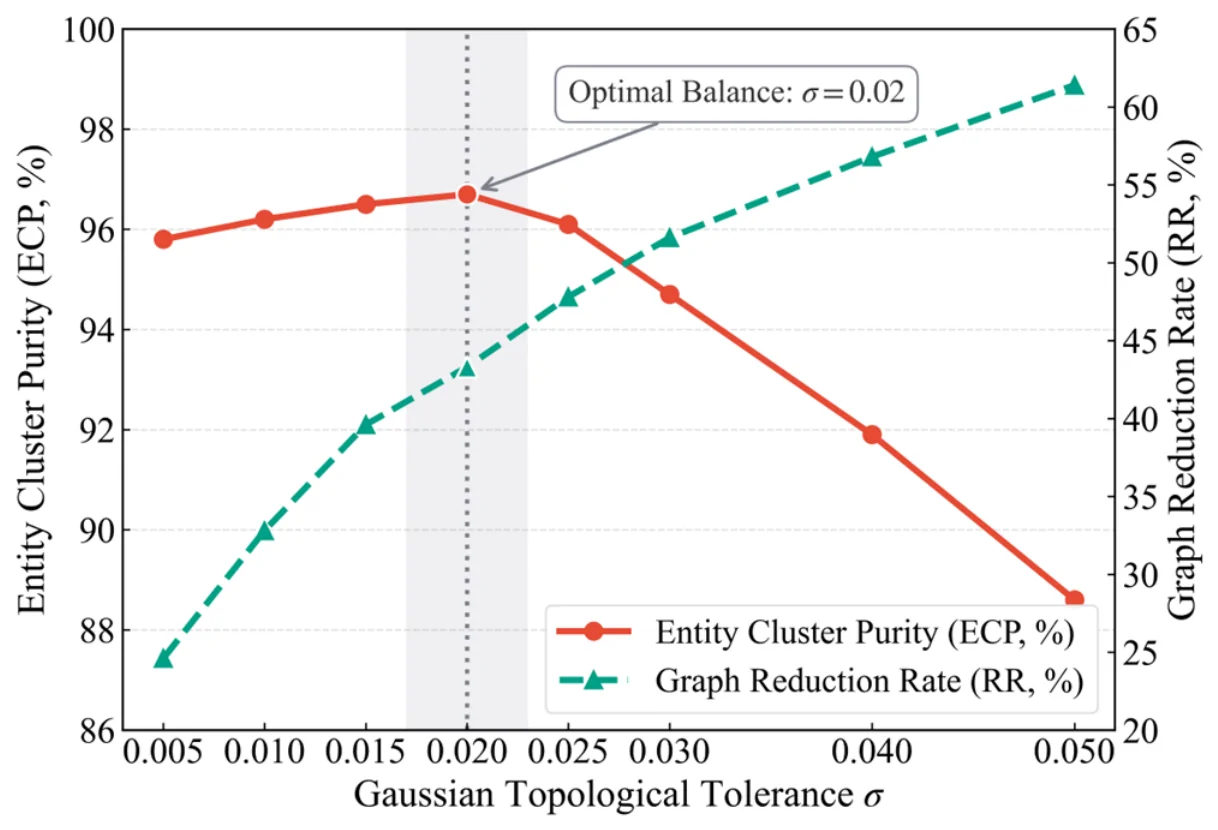
Sensitivity analysis of Gaussian topology tolerance.

As shown in Figure 9, small σ values maintained high *ECP* but produced low *RR*, indicating conservative entity merging and residual graph fragmentation. At *σ* = 0.02, *ECP* reached its maximum value of 96.58%, while *RR* increased to 43.30%. Beyond *σ* = 0.02, *RR* continued to increase while *ECP* progressively decreased, indicating that additional graph compression was accompanied by increasing cross-location merging. These results support the operating point *σ* = 0.02 previously fixed through the joint validation search in Section 4.3, where *σ* represents the tolerance of normalized spatial descriptions. Entity merging additionally requires consistency in component category, semantic context, spatial anchor, and maintenance-event evidence.

### 4.5. Downstream Task Validation and Case Analysis for Fault Traceability

Using the validation-selected value of *σ* = 0.02, downstream fault traceability was evaluated on the independent test set. To verify the practical value of the constructed spatiotemporal knowledge graph in predictive maintenance and fault traceability tasks, an offline fault-replay experiment was designed. The test set contained 175 underlying maintenance events, represented by 175 real seed logs and 525 rule-guided augmented variants. Event-level eligibility screening retained 105 events with complete alarm phenomenon–historical log–spatial location–root-cause chains. The remaining 70 events contained routine-only records, incomplete fault-closure information, or lacked verified gold-standard root-cause nodes. Each retained event comprised one real seed log and three corresponding rule-guided variants, yielding 105 real logs and 315 augmented logs, or 420 fault-replay samples in total. The retained events covered typical industrial scenarios, including roller jamming, coal slurry accumulation, belt deviation, drive motor overload, abnormal pulley vibration, and emergency shutdown events. Each sample contained the final alarm phenomenon, historical maintenance logs, spatial descriptions, and gold-standard root-cause nodes. The downstream task was defined as follows: given a final alarm or fault phenomenon, the system was required to perform reverse retrieval within the knowledge graph and output the Top-1 or Top-3 root-cause candidate nodes, together with the corresponding spatial anchors and causal propagation paths. A prediction was counted as correct only when the predicted root-cause node matched the verified component identity, fault state, and spatial anchor within the predefined tolerance. Predictions with the correct fault type but an incorrect component identity or spatial location were counted as incorrect. Therefore, Accuracy@1, Recall@3, and MRR were calculated at the root-cause-node level rather than at the coarse fault-category level. The three ranking metrics were calculated separately for the 105 real seed logs, the 315 augmented variants, and the combined 420-sample set. Statistical significance testing used the 105 underlying maintenance events as independent resampling clusters.

Five methods were compared in the downstream experiment: Keyword Retrieval, Static Equipment KG, R-GCN [31], Static spatiotemporal knowledge graph without decay (Static STKG without Decay), and Dynamic STKG. The R-GCN baseline contained two relational graph-convolution layers with 128-dimensional hidden representations and a DistMult decoder. It was trained using Adam with a learning rate of 0.001, a dropout rate of 0.2, and ten negative triples per positive triple. Validation-set MRR was used for early stopping. Downstream evaluation used three matched runs. In each run, seed-specific CCAS extraction and entity-alignment outputs were used to construct a run-specific graph. R-GCN was independently initialized and trained on this graph, while Static STKG without Decay and Dynamic STKG were evaluated on the same graph and differed only in temporal confidence decay. For Dynamic STKG, confidence was normalized to [0, 1], with *C*_0_ = 1.0 assigned to newly activated fault-related entities. The archival threshold was fixed at *C*_arch_ = 0.20. The decay constant *λ* was evaluated on the validation-set temporal replay over 0.010–0.030 day^−1^ at intervals of 0.005 day^−1^. Accuracy@1 was used as the primary selection criterion and MRR as the secondary criterion, yielding *λ* = 0.020 day^−1^. These parameters were fixed before test-set evaluation and were used in all three downstream runs and in the temporal-window replay. Keyword Retrieval and Static Equipment KG used fixed rules and fixed graph structures, yielding identical rankings across runs. The evaluation metrics included root-cause localization accuracy (Accuracy@1), Top-3 recall (Recall@3), mean reciprocal rank (MRR), the average rule-based simulated troubleshooting-time estimate, and its estimated reduction relative to Keyword Retrieval. The troubleshooting-time metric was defined as a rule-based simulated estimate rather than an algorithm runtime or a field-measured maintenance duration. For each fault-replay sample, three time costs were calibrated from the conveyor length, spatial access conditions, and standard inspection procedures. *T*_1*,i*_ represents targeted inspection of the Top-1 candidate location, *T*_3*,i*_ represents sequential inspection of up to three candidate locations, and *T*_*full,i*_ represents conventional full-line inspection. Across the 420 replay samples, these costs were 23.95 ± 5.62 min, 87.16 ± 15.77 min, and 224.90 ± 23.97 min, respectively, with corresponding ranges of 12–38 min, 55–125 min, and 170–280 min. The calibration rules and lookup values were fixed before evaluation and applied identically to all comparison methods. The average rule-based simulated troubleshooting-time estimate was calculated over the 420 retained fault-replay samples as follows:

*t_i_* = *T*_1*,i*_, **if** Top-1 correct (25)

*t_i_* = *T*_3*,i*_, **if** Top-3 correct but Top-1 incorrect (26)

*t_i_* = *T*_*full,i*_, **if** Top-3 incorrect (27)

*Avg. Time* = (1/*N*) × ∑_*i*=1_^*N*^
*t_i_* (28)

*Reduction* = (*T_keyword_* − *T_method_*)/*T_keyword_* × 100% (29)

where *N* = 420; *T*_1*,i*_, *T*_3*,i*_, and *T*_*full,i*_ denote the sample-level time costs of targeted inspection, sequential inspection of three candidate locations, and conventional full-line manual inspection for the *i*-th fault-replay sample, respectively; *T_keyword_* denotes the average simulated troubleshooting time of the keyword-retrieval baseline; and *T_method_* denotes the average simulated troubleshooting time of the evaluated method.

The time assignments were calibrated from anonymized historical work orders and standard inspection procedures before evaluation, and were then applied consistently to each retained fault-replay sample according to its ranking result, spatial access condition, and inspection category. The experimental results are presented in Table 9.

As shown in Table 9, Dynamic STKG achieved an Accuracy@1 of 92.78 ± 0.36%, a Recall@3 of 97.38 ± 0.24%, and an MRR of 0.950 ± 0.004. Compared with R-GCN, Dynamic STKG increased Accuracy@1 by 10.16 percentage points, and the improvement over Static STKG without Decay was 6.59 percentage points. The event-cluster-aware paired bootstrap yielded Holm-adjusted *p*-values of 0.011 and 0.032 for the two comparisons, respectively. The external comparison confirms the advantage of the graph framework, while the ablation comparison isolates the contribution of temporal confidence decay. The rule-based simulated troubleshooting-time estimate was 30.40 ± 0.70 min, corresponding to an estimated reduction of 68.50 ± 0.73% relative to Keyword Retrieval. These results indicate stable root-cause ranking and suppression of outdated fault evidence across three independent runs. To characterize downstream performance across data sources, the 420 fault-replay samples were further evaluated separately on the 105 real seed logs and 315 rule-guided augmented variants. The results are shown in Table 10.

**Figure 10:**
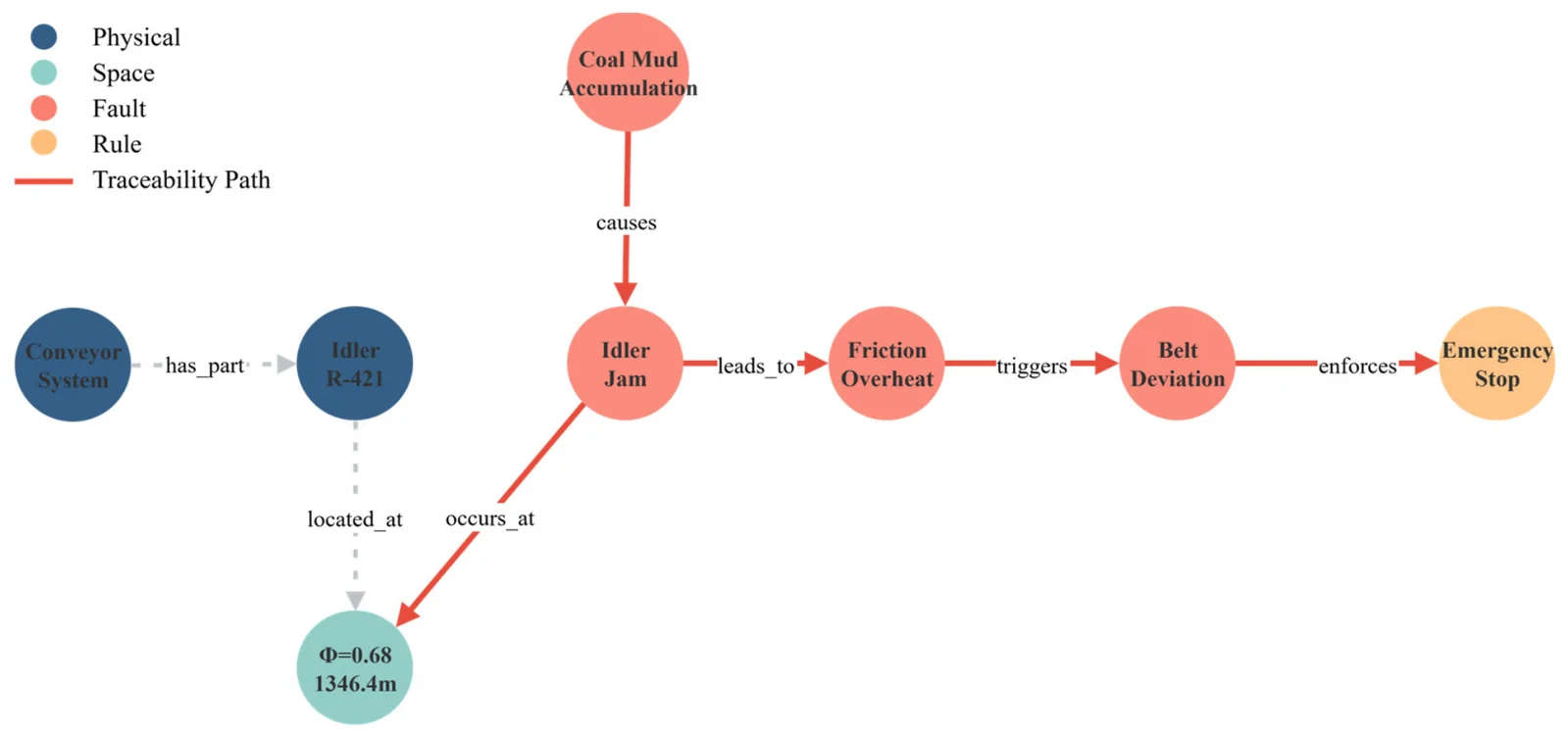
Fault Traceability Topology Graph.

As shown in Table 10, Dynamic STKG achieved an Accuracy@1 of 93.97 ± 0.55%, a Recall@3 of 98.41 ± 0.55%, and an MRR of 0.960 ± 0.002 on the real seed logs. The corresponding results on the augmented variants were 92.38 ± 0.63%, 97.04 ± 0.37%, and 0.947 ± 0.005, respectively. Dynamic STKG retained the highest ranking performance on both subsets, with differences of 1.59 percentage points in Accuracy@1, 1.37 percentage points in Recall@3, and 0.013 in MRR between the two subsets. To validate the practical effectiveness of the constructed spatiotemporal knowledge graph for belt conveyor systems in real industrial scenarios, this section presents a visual representation of the structured four-dimensional ontology network using a graph database. A cross-spatial fault traceability case is analyzed to illustrate the underlying mechanism. As shown in Figure 10, the global topology reveals that physical entity nodes, spatial manifold anchors, fault evolution states, and rigid constraint rules are interconnected through multiple directed edges. These connections form a semantically interpretable network for fault tracing. For the evaluated 1980 m conveyor with a belt width of 1.60 m, alarms involving drive-motor current overload and belt deviation initiate reverse tracing along the spatiotemporal cascade paths. The reasoning process identifies a local friction-overheating node at the normalized spatial anchor Φ = 0.68, corresponding to approximately 1346.40 m from the starting point, and links this location with historical maintenance records describing severe coal-slurry accumulation and roller jamming. Based on these connected nodes, the graph reconstructs the fault-evolution sequence from coal-slurry accumulation through roller jamming and localized friction overheating to belt deviation and the emergency-shutdown response. A location-specific maintenance work order is then generated for the identified spatial region. This case illustrates the combined use of normalized spatial anchoring, causal-path reconstruction, root-cause localization, and location-specific maintenance support within the evaluated conveyor setting.

**Figure 11:**
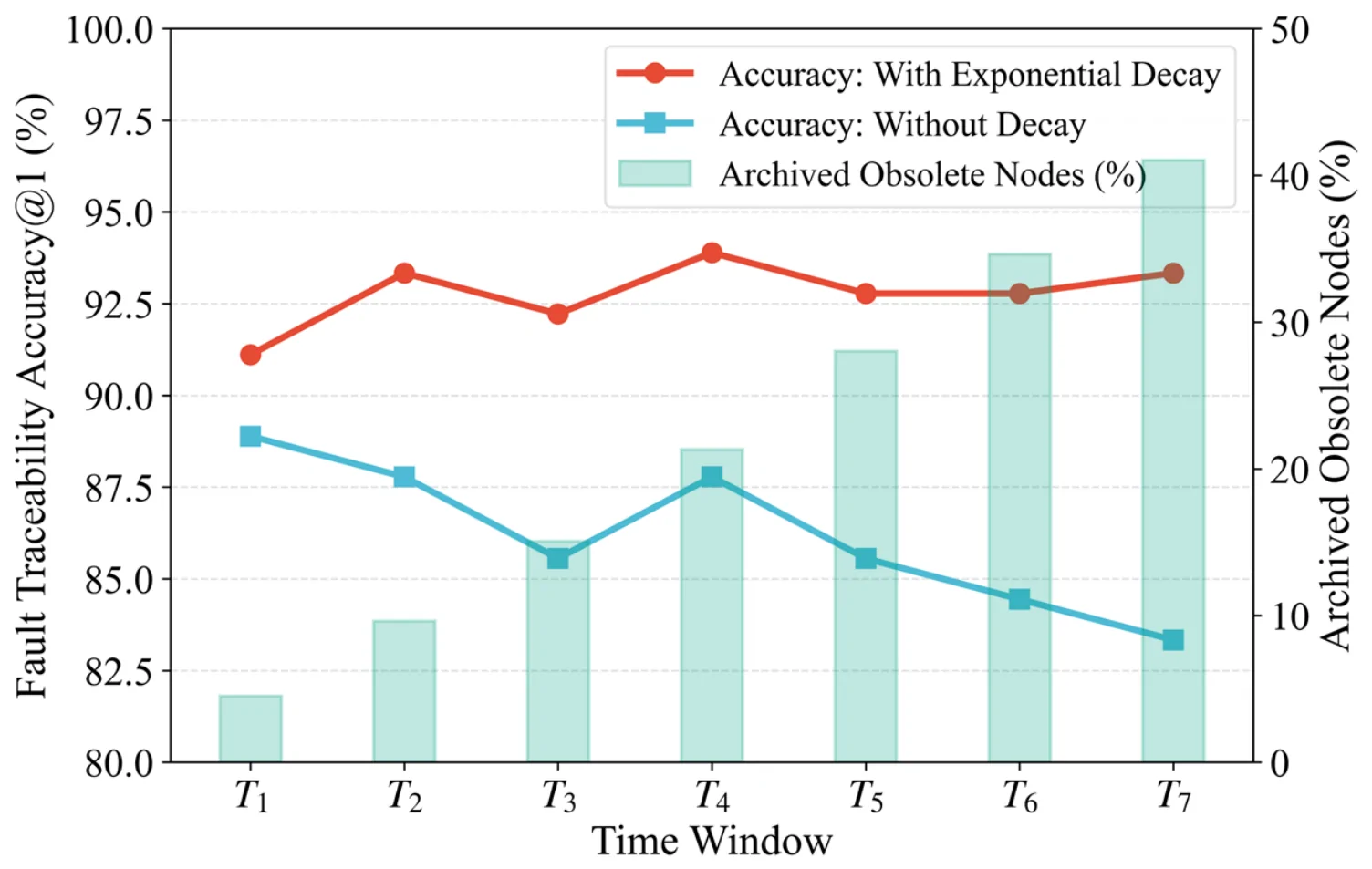
Effect of exponential temporal decay on dynamic graph-based fault traceability.

To isolate the effect of exponential confidence decay, the temporal-window replay analysis was conducted using the CCAS extraction and entity-alignment outputs from one predefined inference run. The 105 retained maintenance events were ordered by maintenance timestamp and divided into seven consecutive, non-overlapping windows of 15 events each. Each window contained 60 textual records, comprising 15 real seed logs and 45 corresponding rule-guided variants. The windows were denoted *T*_1_–*T*_7_ in chronological order, with *T*_1_ representing the earliest events and *T*_7_ representing the latest events. The decay and no-decay settings started from the same initial graph and processed identical replay samples in the same order, with all retrieval rules, ranking parameters, and graph-update settings fixed. Exponential temporal decay was the only difference between the two settings. The results are shown in Figure 11.

Without temporal decay, historically frequent fault nodes retained high confidence and increasingly interfered with the ranking of current root causes, causing Accuracy@1 to show an overall downward trend across successive windows. With temporal decay enabled, the confidence of processed or long-inactive fault nodes gradually decreased. Nodes below the archival threshold were excluded from current-priority reasoning, while the proportion of archived obsolete nodes increased over time. Consequently, the dynamic graph maintained more stable traceability accuracy and reduced interference from outdated fault evidence.

## 5. Conclusions and Future Work

This study addresses spatial ambiguity and unstructured data fusion challenges in knowledge graph construction for long-distance linear industrial assets such as belt conveyor systems. An LLM-driven framework for linear topology-aware spatiotemporal knowledge graph construction is proposed. Experimental results and case analysis lead to the following conclusions:

(1) A Physical–Space–Fault–Rule ontology and normalized linear topology mapping mechanism were developed for long-distance belt conveyor maintenance knowledge graphs. By assigning unique spatial anchors to homogeneous components, the framework improves the spatial distinguishability of linear assets and provides a reusable topological basis for entity disambiguation and fault-causal reasoning.
(2) A CCAS extraction mechanism was constructed to transform noisy maintenance logs into structured fault knowledge. Under the structured gold-standard annotation protocol, the method achieved a global F1-score of 94.10%, demonstrating its ability to extract fault states, causal relations, maintenance actions, and spatial anchors from colloquial industrial text.
(3) The LTA-Alignment algorithm combined Gaussian topological penalties with LLM-based grey-zone arbitration to suppress semantic collapse among homogeneous components. Entity cluster purity increased from 45.20% to 96.70%. In downstream traceability, the dynamic graph achieved an Accuracy@1 of 92.78% and an estimated 68.50% reduction in rule-based simulated troubleshooting time relative to Keyword Retrieval.

The results establish the effectiveness of the proposed framework for the investigated 1980 m non-branching conveyor line and its verified English maintenance-log corpus. Transfer to other enterprises and conveyor layouts requires adaptation of equipment dictionaries, topology mappings, and alignment parameters. Future work will evaluate cross-line transfer, branching conveyor topologies, direct processing of original-language logs, and the extension of the proposed framework from text-centered knowledge extraction to multimodal industrial Internet of Things scenarios. High-frequency vibration signals, infrared thermal images, inspection records, and dynamic knowledge graphs will be integrated through edge–cloud collaborative computing to support real-time graph updating and cross-modal evidence fusion. In addition, semi-supervised validation and automatic consistency checking will be introduced to reduce dependence on expert arbitration in ambiguous annotation cases. This extension is expected to provide a dynamically evolving semantic backbone for industrial digital twins and adaptive predictive maintenance.

## Figures and Tables

**Table 1:** Statistics of the Constructed Dataset.

Dataset Dimension	Value	Description
Total Physical Entities	11,850	Derived from real equipment BOMs, including sub-component breakdowns.
Highly Homonymous Components	2310	Includes 1650 sets of carrying idlers and 660 sets of return idlers with highly overlapping text features.
Spatial Manifold Domain	*L_total_* = 1980 m	Benchmarked against the total length of typical heavy-duty mining main conveyor belts.
Unstructured Log Entries	3500	Augmented based on real maintenance ledgers, containing severe spelling errors and complex semantic noise.
Gold-standard Knowledge Triples	8230	Structured associations constructed through the gold-standard annotation protocol for algorithm benchmarking.

**Table 2:** Experimental configuration and parameter settings.

Category	Parameter	Value	Function
Data partition	train/validation/test	7:1:2	Fixed data partition
LLM inference	temperature	0.1	Reduce randomness
LLM inference	top-p	0.9	Control sampling range
LLM inference	max tokens	1024	Limit output length
LLM output	Format	Constrained JSON	Enforce structured outputs
Output stability	inference runs	3	Repeated inference for each log
LTA-Alignment	*τ_low_*	0.55	Direct rejection for low scores
LTA-Alignment	*τ_high_*	0.75	Direct merging for high scores
LTA-Alignment	*σ*	0.02	Control spatial tolerance
Dynamic STKG	Initial confidence *C*_0_	1.0	Initialize fault-state confidence
Dynamic STKG	Decay constant *λ*	0.020 day^−1^	Control temporal confidence decay
Dynamic STKG	Archival threshold *C*_arch_	0.20	Archive low-confidence historical fault states
Alignment evaluation	Candidate pairs	12,600	Evaluate alignment performance
Downstream evaluation	test logs	700 (175 event clusters)	175 real seed logs and 525 augmented variants
Downstream evaluation	traceability samples	420 (105 event clusters)	105 real seed logs and 315 augmented variants with complete root-cause chains

**Table 3:** Representative gold-standard annotation examples for maintenance-log evaluation.

Evaluation Task	Raw Evidence	Gold-Standard Label	Matching Criterion
CCAS extraction	“At approximately 1000 m, the belt deviated to the right. Inspection found that a nearby roller was jammed by coal slurry, and the shell temperature was high. Shutdown handling was required on site.”	Condition: roller jamming; frictional heating; belt deviation. Component: carrying idler. Causal chain: coal slurry accumulation → roller jamming → frictional heating → belt deviation. Action: shutdown handling. Space: Φ = 1000/1980 ≈ 0.505.	The extracted structured fields must match the gold-standard CCAS fields. The normalized spatial anchor is correct only when the coordinate error is ≤0.02.
Entity alignment	BOM entity: “Carrying Idler #R-053 at Φ = 0.455”. Log entity: “roller with abnormal noise near 900 m”.	Merge label: positive. Both records refer to the same carrying idler under consistent equipment category, spatial anchor, and maintenance context.	The candidate pair is counted as correctly merged only when component category, spatial anchor, and maintenance-event evidence support the same physical entity.
Entity rejection	Entity A: “carrying idler near Φ = 0.455”. Entity B: “carrying idler near Φ = 0.780”.	Merge label: negative. The two entities have similar names but correspond to different physical locations.	The pair must be rejected when spatial anchors indicate different physical components, even if their textual descriptions are semantically similar.
Root-cause tracing	Final alarm: belt deviation and motor overload. Historical evidence: coal slurry accumulation and roller jamming near Φ = 0.505.	Root-cause node: coal-slurry-induced roller jamming near Φ = 0.505.	A prediction is correct only when the returned root-cause node matches component identity, fault state, and spatial anchor within the predefined tolerance.

**Table 4:** Core prompt templates for CCAS extraction and LLM-based grey-zone arbitration.

Module	Prompt Template
CCAS Knowledge Extraction	You are an expert in belt conveyor maintenance knowledge graph construction. Given an inspection log, perform extraction through the following ordered steps: (1) normalize colloquial and non-standard expressions and identify the abnormal condition; (2) identify the physical component and apply equipment-topology constraints to resolve its identity; (3) reconstruct the directional fault-evolution relation; (4) identify the maintenance action; and (5) recognize the raw location and calculate the normalized spatial anchor Φ ∈ [0, 1]. Return only valid JSON with the fields condition, component, fault_state, causal_chain, action, raw_location, normalized_phi, and confidence. Do not output intermediate reasoning or additional explanations.
Spatial Anchoring	Convert the raw location expression into a normalized spatial coordinate. If the text contains absolute distance l, compute Φ = *l*/*L_total_*. If the text contains only relative location, infer the most likely range according to the conveyor topology and return a confidence score.
Grey-Zone Arbitration	You are a maintenance engineer checking whether two candidate entities refer to the same physical component. Given semantic similarity, spatial deviation, timestamps, and maintenance context, decide MERGE or REJECT. Consider whether the two records describe a continuous causal process of the same fault. Return JSON: decision, reason, confidence.

**Table 5:** Run-averaged results on the real, augmented, and combined test subsets.

Test Subset	No. of Logs	Precision (%)	Recall (%)	Global F1 (%)	*ECP* (%)	*RR* (%)
Real seed logs	175	95.32	94.68	95.00	97.12	44.60
Rule-guided augmented logs	525	94.21	93.47	93.84	96.47	45.10
Combined test set	700	94.50	93.80	94.10	96.70	45.30

**Table 6:** Field-level evaluation units of CCAS extraction.

Evaluation Unit	Matching Criterion
Condition	Correct abnormal state or operating condition is extracted
Component	Component category and entity mention are correctly identified
Fault state	Fault mode or degradation state matches the gold-standard label
Causal relation	Directional causal chain is consistent with the gold-standard relation
Maintenance action	Handling action or maintenance decision is correctly extracted
Raw location	Original location expression is correctly recognized
Normalized spatial anchor	Φ error ≤ 0.02

**Table 7:** Comparison with the external entity-alignment method.

Method	Merge Precision (%)	Merge Recall (%)	Merge F1 (%)	*ECP* (%)	*RR* (%)
SCSA [23]	91.60 ± 0.50	93.20 ± 0.61	92.39 ± 0.56	92.80 ± 0.62	49.70 ± 0.66
LTA-Alignment	96.40 ± 0.51	95.10 ± 0.54	95.75 ± 0.53	96.70 ± 0.53	45.30 ± 0.37

**Table 8:** Contextual arbitration of a representative grey-zone entity pair with spatial-estimation uncertainty.

Feature Dimension	Case Data & LLM Input Context
Candidate Entities	*e_i_*: (BOM) Carrying Idler #R-053 at Φ = 0.455; *e_j_*: (Log) “Roller with abnormal noise” at Φ = 0.473.
Numerical Scores	*Sim_sem_* = 0.980 (High); *Sim_top_* = 0.667 (Penalized due to *δ* = 0.018 deviation); *S_align_* = 0.654 (Grey-Zone).
Arbiter Input Context	Maintenance Log A (10:00 a.m.): “Worker A reported R-053 making clicking sounds near the 900 m mark.” Maintenance Log B (11:30 a.m.): “Worker B found the roller at ~936 m was jammed by coal mud and replaced its bearing.”
Contextual Arbitration Evidence	Both records describe a carrying idler, with *S_sem_* = 0.980. The normalized spatial deviation is *δ* = 0.018, yielding *S_top_* = 0.667 and *S_align_* = 0.654. The abnormal-noise report precedes the jamming diagnosis. Bearing replacement completes the fault-handling sequence and links the records within the same maintenance event.
Final Decision	Decision: MERGE. The consistent component category, temporal sequence, fault-state evolution, and bearing-replacement action support the same physical entity under spatial-reporting uncertainty.

**Table 9:** Downstream root-cause traceability results in the offline fault-replay experiment.

Method	Acc@1 (%)	Rec@3 (%)	MRR	Avg. Time (min)	Reduction (%)
Keyword Retrieval	54.76 ± 0.00	68.10 ± 0.00	0.610 ± 0.000	96.50 ± 0.00	0.00 ± 0.00
Static Equipment KG	71.43 ± 0.00	82.62 ± 0.00	0.780 ± 0.000	68.20 ± 0.00	29.33 ± 0.00
R-GCN [31]	82.62 ± 0.95	90.48 ± 0.71	0.870 ± 0.006	49.90 ± 0.62	48.29 ± 0.64
Static STKG without Decay	86.19 ± 0.71	93.10 ± 0.71	0.900 ± 0.005	43.70 ± 0.53	54.72 ± 0.55
Dynamic STKG	92.78 ± 0.36	97.38 ± 0.24	0.950 ± 0.004	30.40 ± 0.70	68.50 ± 0.73

**Table 10:** Downstream root-cause traceability performance on the real seed logs and rule-guided augmented variants.

Method	Real Acc@1 (%)	Real Rec@3 (%)	Real MRR	Augmented Acc@1 (%)	Augmented Rec@3 (%)	Augmented MRR
Keyword Retrieval	56.19 ± 0.00	69.52 ± 0.00	0.621 ± 0.000	54.29 ± 0.00	67.62 ± 0.00	0.606 ± 0.000
Static Equipment KG	73.33 ± 0.00	82.86 ± 0.00	0.790 ± 0.000	70.79 ± 0.00	82.54 ± 0.00	0.777 ± 0.000
R-GCN [31]	84.13 ± 1.10	91.75 ± 1.45	0.881 ± 0.007	82.12 ± 0.97	90.05 ± 0.48	0.866 ± 0.006
Static STKG without Decay	86.35 ± 1.10	93.97 ± 0.55	0.908 ± 0.002	86.14 ± 0.66	92.80 ± 0.80	0.897 ± 0.006
Dynamic STKG	93.97 ± 0.55	98.41 ± 0.55	0.960 ± 0.002	92.38 ± 0.63	97.04 ± 0.37	0.947 ± 0.005

## Data Availability

The data and code supporting the findings of this study are available from the corresponding author upon reasonable request. Public release is currently restricted due to ongoing research and data-use constraints associated with enterprise maintenance records.
